# Pan-cancer adaptive immune resistance as defined by the Tumor Inflammation Signature (TIS): results from The Cancer Genome Atlas (TCGA)

**DOI:** 10.1186/s40425-018-0367-1

**Published:** 2018-06-22

**Authors:** Patrick Danaher, Sarah Warren, Rongze Lu, Josue Samayoa, Amy Sullivan, Irena Pekker, Brett Wallden, Francesco M. Marincola, Alessandra Cesano

**Affiliations:** 1 NanoString Technologies Inc, Seattle, WA USA; 2 AbbVie Inc., Redwood City, CA USA

**Keywords:** Checkpoint inhibition, Tumor inflammation signature (TIS), The Cancer Genome Atlas (TCGA), Gene signature

## Abstract

The Tumor Inflammation Signature (TIS) is an investigational use only (IUO) 18-gene signature that measures a pre-existing but suppressed adaptive immune response within tumors. The TIS has been shown to enrich for patients who respond to the anti-PD1 agent pembrolizumab. To explore this immune phenotype within and across tumor types, we applied the TIS algorithm to over 9000 tumor gene expression profiles downloaded from The Cancer Genome Atlas (TCGA). As expected based on prior evidence, tumors with known clinical sensitivity to anti-programmed cell death protein 1 (PD-1) blockade had higher average TIS scores. Furthermore, TIS scores were more variable within than between tumor types, and within each tumor type a subset of patients with elevated scores was identifiable although with different prevalence associated with each tumor type, the latter consistent with the observed clinical responsiveness to anti PD-1 blockade. Notably, TIS scores only minimally correlated with mutation load in most tumors and ranking tumors by median TIS score showed differing association to clinical sensitivity to PD-1/PD-1 ligand 1 (PD-L1) blockade than ranking of the same tumors by mutation load. The expression patterns of the TIS algorithm genes were conserved across tumor types yet appeared to be minimally prognostic in most cancers, consistent with the TIS score serving as a pan-cancer measurement of the inflamed tumor phenotype. Characterization of the prevalence and variability of TIS will lead to increased understanding of the immune status of untreated tumors and may lead to improved indication selection for testing immunotherapy agents.

**Electronic supplementary material:**

The online version of this article (10.1186/s40425-018-0367-1) contains supplementary material, which is available to authorized users.

## Background

Recognition of the importance of the tumor immune microenvironment in suppressing antitumor immunity has led to significant advances in tumor immunotherapy. Treatments are now available that overcome tumor cells’ ability to evade immune detection and harness the “non-self”-directed specificity of the immune system to attack tumors across multiple cancer types. In addition, immunotherapies, unlike cytotoxic or targeted therapies, have the advantage of triggering a memory immune response that clinically manifests in specific, systemic, and durable antitumor effect.

Among the most promising therapeutic approaches to re-activating anti-tumor immunity is the pharmacological manipulation of physiologic immune checkpoints. Immune checkpoints refer to inhibitory pathways in the immune system crucial for maintaining self-tolerance and minimizing the possibility of chronic autoimmune inflammation. Exploitation of immune checkpoint pathways is a major mechanism by which tumors escape immune surveillance, and immune checkpoint blockade is the basis for the clinical anti-tumor activity of most of the currently approved immuno-oncology agents targeting CTLA-4 (ipilumimab) and programmed cell death protein 1 (PD-1) (nivolumab, pembrolizumab,) or PD-1 ligand 1 (PD-L1) (atezolizumab, durvalumab, and avelumab) [1].

Despite this progress, only a minority of patients with advanced/metastatic cancer respond to immune checkpoint inhibitors, thus exposing the remaining patients to potentially ineffective, toxic, and costly treatments. Therefore, biomarkers predicting response are needed to guide treatment decisions in the clinic and to enable clinical trials to succeed in populations where response is rare.

In this regard, increased PD-L1 expression (as measured by immunohistochemistry [IHC]) on the surface of tumor cells and/or immune cells, despite representing today the only form of approved companion diagnostics for immunotherapies targeting the PD-1 axis, has been shown to be only inconsistently associated with these agents’ clinical benefit [1]. This may be due to limitations intrinsic to the analyte measured (i.e., significant cellular, spatial, and temporal heterogeneity) and the platform used (i.e., subjective interpretation) [2]. In addition, the drug-centric approach of independently developing a PD-L1 IHC assay for each anti-PD-1/PD-L1 agent has resulted in a lack of “gold standard” assay, complicating testing and decision making in the clinic.

Additional predictive biomarkers have been investigated for use in immuno-oncology. For example, abundance and location of tumor infiltrating lymphocytes has been proposed as biomarker [3]. The most advanced assay in use to date is the Immunoscore, an immunohistochemistry based assay which quantitates abundance and phenotype of T cells [4]. The Immunoscore has been shown to be highly prognostic in colorectal cancer (CRC) [5], but its utility as a predictive marker remains uncharacterized.

More recently, clinical trial data have demonstrated the utility of measuring microsatellite instability (MSI) status and/or DNA mismatch repair deficiency (dMMR) as predictive markers for response to PD-1 blockade independently from tumor cell of origin, resulting in the first FDA pan-cancer approval of a therapeutic in oncology (pembrolizumab) [6]. The association of response to PD-1 blockade in dMMR tumors was first observed in a single patient with MSI-hi CRC in the nivolumab trial MDX1106–03 [7]. This initial result was then extended to show that patients with dMMR tumors experiences 27% ORR in CRC and 43% ORR in non-CRC after receiving pembrolizumab, demonstrating the predictive power of biomarker [8]. Recently, pembrolizumab has received FDA approval in all indications where a tumor has dMMR, although the companion diagnostic remains undefined. Unfortunately, dMMR occurs in approximately 5% of CRC and endometrial tumors, and is much less frequent in other indications.

Another approach to characterizing potential neoantigen load that can be applied to a wider spectrum of tumors is measurement of total tumor mutation burden (TMB). The earliest successes of checkpoint inhibitors were in melanomas and non-small cell lung cancers, two tumors that can have high mutation burden due to mutagen exposure (UV light and tobacco smoke). The correlation of TMB and response to checkpoint inhibitors was first demonstrated in lung cancer, which has a broad range of nonsynonymous mutations within the tumor [9]. Since then, it has been demonstrated that tumor types with higher median mutation burden tend to be more response to checkpoint inhibitors than tumors that harbor few mutations [10]. Today, a number of platforms to detect TMB are being developed for routine clinical application, most prominently the FoundationOne assay, which reports on mutation status of 324 genes [11].

Because of the complexity of tumor-immune interactions, efforts to capture this complexity via a single analyte such as PD-L1 expression as measured by IHC, or tumor mutation load as a surrogate of potential tumor antigenicity, yields limited and incomplete information about the complex and dynamic nature of the tumor-immune microenvironment.

More recently, gene expression in the tumor microenvironment, using RNA isolated from formalin-fixed paraffin-embedded (FFPE) pretreatment samples from patients undergoing anti-PD-1/PD-L1 pathway treatment have been described [12–14]. These signatures measure, using various technology platforms, different but highly correlated gene transcripts associated with the presence of an adaptive immune response that is peripherally suppressed, a phenotype that appears to be necessary, although not sufficient, for clinical benefit from PD-1/PD-L1 blockade. One of these signatures, described by Ayers et al. (2017) [12], was developed on the NanoString nCounter gene expression system (NanoString Technologies, Inc., Seattle, WA) in the context of pembrolizumab treatment as a pan-tumor determinant of response to PD-1-directed therapy. Samples were obtained at baseline from patients undergoing treatment with pembrolizumab in clinical trials of multiple distinct tumor types in a rigorous stepwise validation of the hypothesis that immune-related gene signatures can enrich for clinical response to PD-1 checkpoint blockade, including samples from KEYNOTE-001, KEYNOTE-006, and KEYNOTE-028. The final analytically validated, IUO-ready gene expression signature, named the Tumor Inflammation Signature (TIS), contains genes related to antigen presentation, chemokine expression, cytotoxic activity, and adaptive immune resistance (Table 1). A score is calculated as a weighted linear combination of the 18 genes’ expression values normalized to stable housekeeper gene expression, and scores above a fixed threshold can be used to evaluate patients whose tumor would benefit from pembrolizumab administration. The TIS has been developed into a clinical trial assay running on the nCounter Analysis System which has been applied retrospectively in multiple immuno-oncology trials (KEYNOTE-180, KEYNOTE-181, KEYNOTE-158).

**Table 1:** Genes in the Tumor Inflammation Signature

TIS Biology	Gene	Protein	Function
Antigen Presenting Cell Abundance	PSMB10	PSB10	Immunoproteosome Subunit
	HLA-DQA1	MHC class II DQA1	MHC Class II Antigen Presentation
	HLA-DRB1	MHC class II DRB1	MHC Class II Antigen Presentation
	CMKLR1	CML1	Chemokine Receptor
T Cell/ NK Cell Abundance	HLA-E	HLAE	Nonclassical Class I Antigen Presentation
	NKG7	NKG7	Cytolytic Granule Protein
	CD8A	CD8A	MHC Class I Coreceptor
IFN Activity	CCL5	CCL5	Monocytes and Memory T cells Chemoattractant
	CXCL9	CXCL9	Lymphocyte Chemoattractant
	CD27	CD27	Lymphocyte Activation
	CXCR6	CXCR6	T cell Activation
	IDO1	IDO	Inhibitor of T cell Proliferation and Function
	STAT1	STAT1	Transcription Factor Mediating IFN Response
T Cell Exhaustion	TIGIT	TIGIT	Inhibitor of T cell Function
	LAG3	LAG3	Inhibitor of T cell Function
	CD274	PD-L1	Inhibitor of T cell Function
	PDCD1LG2	PD-L2	Inhibitor of T cell Function
	CD276	B7-H3	Inhibitor of T cell Function

As TIS is a measure of pre-existing adaptive immunity that has been peripherally suppressed, we sought to explore how this immune phenotype distributes within and across tumor types, and how it correlates with other relevant variables such as mutation load, other gene expression signatures, and clinical outcomes in the absence of specific immune therapeutic intervention. For this purpose, we applied the TIS algorithm to gene expression data from The Cancer Genome Atlas (TCGA) database of primary tumors. The specific objectives of this study were a) to explore the distribution of TIS scores within and across a wide range of immunotherapy-naive primary tumors; b) to assess the TIS score’s prognostic value; c) to evaluate the association between TIS score and mutation load; d) to contrast TIS scores with expression levels of immune checkpoint molecules targeted by current immuno-oncology drugs in development; and e) to identify gene expression patterns associated with low TIS score (i.e., “cold” tumors). Similar efforts to characterize presence and activity of the intratumoral immune response have been undertaken in the past [15–17], and this report now extends upon those findings by applying the signature which is the basis for a clinical assay across a spectrum of both solid and hematological malignancies.

## Experimental section

### TCGA data download

Level 3 RSEM-normalized RNASeqV2 data and level 3 mutation packager calls were downloaded from TCGA database. Per standard practice and in alignment with the TIS algorithm, RNASeq data were log2-transformed to avoid extremely skewed gene expression distributions and to allow additive methods like linear regression to model fold-changes rather than absolute expression increases. Each patient’s mutation load was calculated as the number of non-synonymous mutations and then log2-transformed before analysis.

Statistical methods:

- Calculation of TIS score

To maximize fidelity of our computational TIS score calculation to the clinical nCounter TIS assay, we re-normalized the RSEM RNAseq data using the 10 reference (“housekeeping”) genes used in the nCounter assay and performed a log2-transformation of the normalized values. Second, we computed TIS score as a linear combination of the 18 algorithm genes, calculating TIS =${\sum}_{i=1}^{18}{x}_i{w}_i$, where x_i_ is the i^th^ gene’s log2-transformed, normalized expression level and w_i_ is a predefined weight derived in Ayers et al. (2017) [12]. We applied the TIS algorithm to 9083 samples from 32 TCGA RNASeq datasets (Table 2).

b. Association between TIS scores and overall survival

**Table 2:** TCGA Datasets Evaluated

Symbol	N	Name
ACC	79	Adrenocortical carcinoma
BLCA	396	Bladder urothelial carcinoma
BRCA	1092	Breast invasive carcinoma
CESC	301	Cervical squamous cell carcinoma and endocervical adenocarcinoma
CHOL	36	Cholangiocarcinoma
COAD	280	Colon adenocarcinoma
DLBC	47	Lymphoid neoplasm diffuse large B-cell lymphoma
ESCA	183	Esophageal carcinoma
GBM	167	Glioblastoma multiforme
HNSC	516	Head and neck squamous cell carcinoma
KICH	65	Kidney chromophobe
KIRC	530	Kidney renal clear cell carcinoma
KIRP	270	Kidney renal papillary cell carcinoma
LAML	163	Acute myeloid leukemia
LGG	506	Brain lower grade glioma
LIHC	361	Liver hepatocellular carcinoma
LUAD	497	Lung adenocarcinoma
LUSC	483	Lung squamous cell carcinoma
MESO	87	Mesothelioma
OV	264	Ovarian serous cystadenocarcinoma
PAAD	179	Pancreatic adenocarcinoma
PCPG	184	Pheochromocytoma and paraganglioma
PRAD	493	Prostate adenocarcinoma
READ	95	Rectum adenocarcinoma
SARC	249	Sarcoma
SKCM	463	Skin cutaneous melanoma
STAD	409	Stomach adenocarcinoma
TGCT	139	Testicular germ cell tumors
THCA	504	Thyroid carcinoma
THYM	118	Thymoma
UCS	57	Uterine carcinosarcoma
UVM	80	Uveal melanoma

In each cancer type’s dataset, a univariate Cox proportional hazard model was fit predicting overall survival from continuous TIS score. Kaplan-Meier curves were drawn using the R library ggsurvplot.

c. Association between transcriptome and TIS scores

The R library GSA [18] was used to compute the extent of positive and negative association between Gene Ontology (GO) terms [19] and the TIS scores. GO term gene lists were obtained from the Molecular Signatures Database (MsigDb) [20]. The GSA procedure was applied separately to each TCGA dataset taking the input of the dataset’s normalized, log2-transformed expression values as the predictor matrix, the dataset’s TIS scores as the outcome, and GO terms as gene sets.

## Results

### TIS scores are highly variable across and within tumor types, and a subset of patients with elevated scores exists within each tumor type

**Fig. 1:**
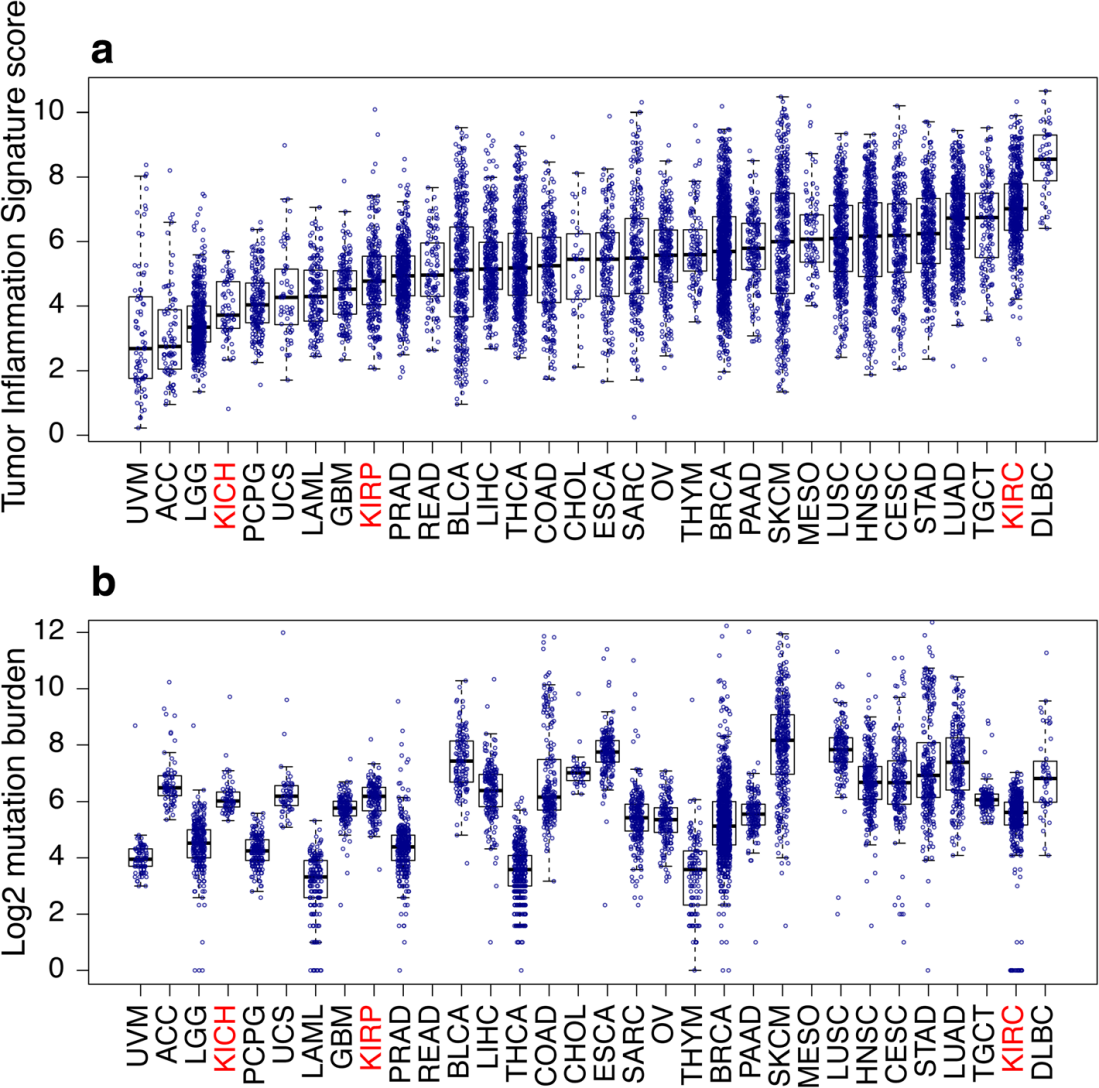
TIS scores in all TCGA patients. **a** Boxplots and points show summary statistics and individual values of TIS scores in each cancer type, ranked by median TIS scores. **b** Boxplots of log2 mutation burden, showed based on ranking in (**a**)

Figure 1 shows TIS scores for all TCGA patients included in the analysis, with tumor types ordered by median TIS scores. While median TIS scores are higher in tumor types with higher rates of response to PD-1/PD-L1 inhibitors (e.g., melanoma, renal cell cancer), and cancers with high mutation load (e.g., non-small cell lung cancer [NSCLC]), within each tumor type there is considerable inter-sample variability. This finding is consistent with a wide range of pre-existent adaptive immunity levels within different tumor types, and it raises the possibility that TIS scores may identify rare responders within tumor types that have low immunotherapy response rates, low median TIS scores, and low mutation burden.

In the clinical setting, in order to use the TIS score as a patient enrichment tool (i.e., identifying “hot” versus “cold” tumors), one or more pre-specified thresholds are needed. Based on TCGA TIS scores, the following thresholds, in diminishing order of stringency, could be reasonable choices depending on the clinical population and the competing desires for enrichment and broad selection: 6.72 is the median TIS score in lung adenocarcinomas and the 75th percentile of all of TCGA; 6.0 is the median TIS score in melanomas and the 61st percentile of all of TCGA; and 5.5 is the median TIS score in all TCGA datasets. Given an estimated offset of 1.3 score units between TIS scores in TCGA and in the IUO assay, these thresholds translate to approximately 8.02, 7.3 and 6.8 in the TIS IUO assay.

Checkpoint inhibitors currently have wider use in late-stage tumors than in first-line settings. Thus the distribution of anti-tumor immunity as measured by TIS score in only late-stage tumors is of interest. Additional file 1: Figure S7 replots (Fig. 1) using only patients with stage IV disease. Within tumor types where sufficient stage IV patients were available, (Fig. 1)‘s ranking of tumor types by median TIS is broadly preserved in stage IV patients, as is the observation of great variability of TIS within all tumor types.

### Tumors with a high TIS score have shown clinical response to anti-PD-1 blockade

Many of the tumor types with high median TIS values, in particular advanced/metastatic renal clear cell carcinoma [21], melanoma [22], lung tumors [23], and head and neck tumors [24], have shown clinical sensitivity to anti-PD-1 blockade (Additional file 2: Figure S1).

The kidney cancers’ responsiveness to checkpoint blockade is well-predicted by TIS scores, but not by mutation burden (Fig. 1, highlighted in red text). Renal clear cell carcinoma (KIRC), an immunogenic tumor type in which immunotherapies such as IL-2 [25], IFN-α [26], and nivolumab [27] have shown clinical benefit in a subset of patients, had the second-highest median TIS scores but fairly low mutation load. In contrast, chromophobe renal cell carcinoma (KICH) and kidney renal papillary cell carcinoma (KIRP), which so far have shown less evidence of susceptibility to anti-PD-1/PD-L1 blockade, have low-ranking median TIS scores and similar median mutation burdens than clear cell carcinomas. Randomized trials investigating the performance of PD1 blockade in these tumors have not been reported to date, but anecdotal data suggests that response rates in the chromophobe subtype, which has the lowest median TIS of the 3 subtypes, has been particularly poor [28, 29]. The finding of a high median TIS score in renal clear cell carcinomas and the efficacy of PD-1 blockade in this tumor type are consistent with other findings that the immunogenicity of renal clear cell carcinoma tumors cannot be explained solely by mutation or neoantigen load, but is highly correlated with MHC class I antigen presenting machinery expression [30].

Furthermore, some tumor types with a moderate TIS score (e.g., pancreatic tumors) have shown notably poor response to immunotherapy in an unselected population. These tumors are known to be highly infiltrated with myeloid cells which may act as external suppressors of anti-tumor immune responses that are not relieved by PD-1 checkpoint blockade [31], raising the possibility of future gene signatures that may by combined with TIS to further dissect immune responses.

### TIS scores are minimally correlated with mutation load within most cancer types

**Fig. 2:**
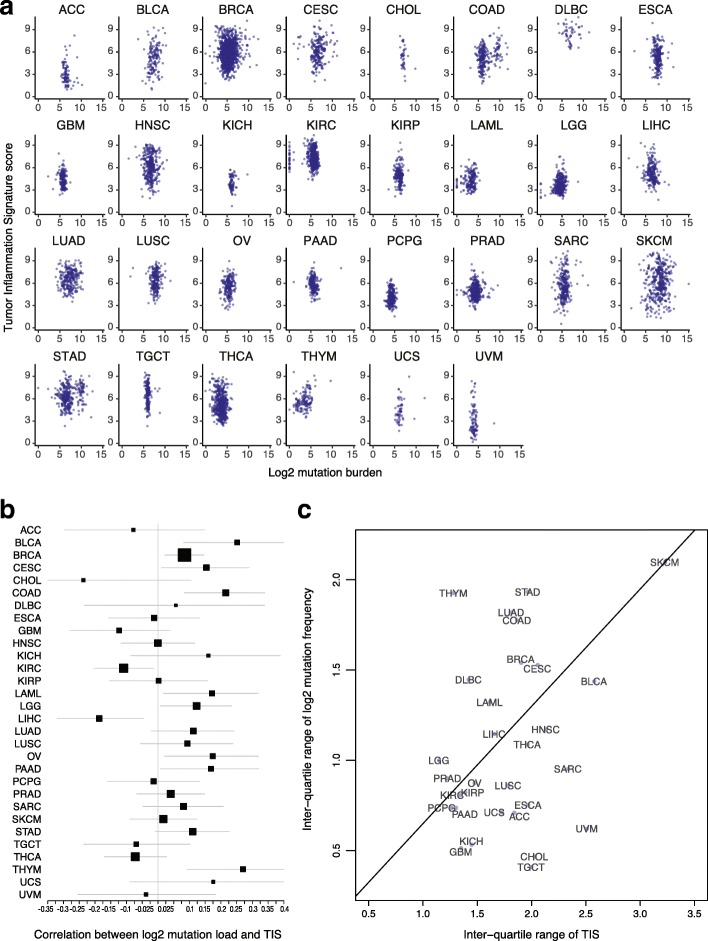
Association between TIS scores and mutation load. **a** TIS score plotted against log2 mutation within each tumor type. **b** Point estimates and 95% confidence intervals for the correlation between TIS score and log2 mutation load within each tumor type. Box size represents the precision of the estimate with larger boxes indicating smaller standard errors; horizontal lines represent 95% confidence intervals. **c** Interquartile range of TIS score and mutation load in each cancer type. To place cancer types in context, a line connects SKCM to the origin

Both TIS scores and mutation load have been investigated as predictive biomarkers for benefit of checkpoint inhibitors. These 2 biomarkers are weakly correlated Fig. 2a: the absolute value of their correlation is below 0.3 in all TCGA datasets, and in most TCGA datasets the 95% confidence interval for their correlation includes 0 Fig. 2b. The strongest correlations between TIS scores and log2 mutation load occurred in colon cancer, which is known to have a hypermutated and immunogenic microsatellite instability high (MSI-H) subtype, and in thymomas and bladder cancer. Additional file 3: Figure S6 shows MSI-H status to be associated with elevated TIS scores in colon and stomach cancers. Liver hepatocellular carcinomas and kidney renal clear cell carcinoma showed statistically significant negative correlations between these biomarkers. A significant fraction of hepatocellular carcinomas are associated with hepatitis B or C virus infection, which may drive inflammation without the presence of a high number of mutations. There is negligible correlation between average mutation burden and average TIS scores across tumor types in TCGA datasets Fig. 2.

### The variability of TIS and mutation load are potential indicators of their predictive utility within cancer types

The predictive utility of TIS and mutation load within a given cancer type can only be definitively established by clinical trials with sample sizes large enough to profile response rates conditional on varying levels of both biomarkers. Until such data is available, large datasets like TCGA can offer important insights into the probable utility of these biomarkers in different cancer types.

A biomarker’s predictive strength in a cancer depends on both the strength of its association with response and its variability. In particular, a biomarker that varies little within a population is less likely to successfully divide that population between responders and non-responders. Melanoma has a prominent role in the literature supporting TIS score and mutation load as predictors of response to checkpoint inhibitors; it also has a higher variability of both biomarkers than any other cancer type Fig. 2c. Some cancer types retain most of melanoma’s TIS score variability while losing much greater variability in mutation load Fig. 2c, below the line, while others retain a greater proportion of melanoma’s mutation load variability than its TIS score variability Fig. 2c, above the line. Since a biomarker’s predictive strength in a cancer depends in part on its variability, Fig. 2c can support educated guesses on the performance of each putative predictive biomarker in any given tumor type (in the context of PD-1/PD-L1 pathway blockade). For example, the interquartile range of mutation load in lung squamous carcinoma (LUSC), head and neck squamous cell carcinoma (HNSC), and sarcomas (SARC) is approximately half as large as its interquartile range in SKCM, suggesting that the predictive utility of mutation burden observed in melanoma will be lower in these cancers. Therefore, in these tumor types and in the context of pharmacological blockade of the PD-1/PD-L1 pathway, TIS score may be a more useful predictive biomarker. Conversely, adenocarcinomas of the lung (LUAD), colon (COAD), and stomach (STAD) have high mutation burden variability but lower TIS variability than seen in melanomas. However, since biomarker utility also depends on how closely related the biomarker is to the mechanism of action of the drug, and since TIS measures transcriptional activity in the tumor microenvironment directly related to immune adaptive resistance, the TIS may provide additional utility in the context of mutation load, which is measuring potentially immune activating neoantigen expression, to enrich for clinical response to anti-PD1/PD-L1.

### Evaluation of prognostic value of TIS scores

**Fig. 3:**
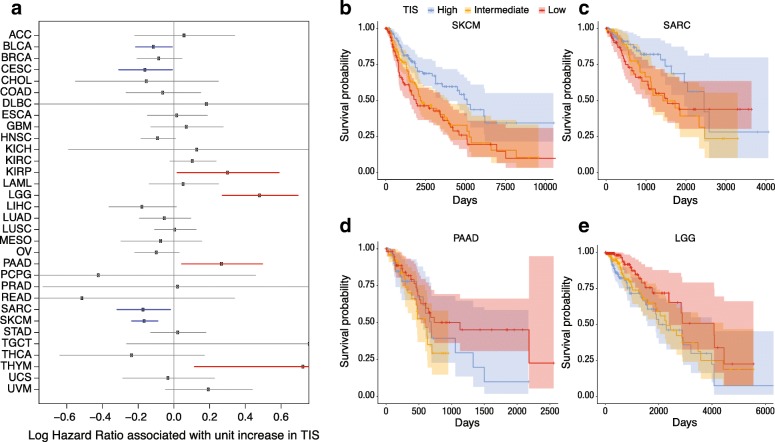
Association between TIS score and overall survival in TCGA. **a** Forest plot showing log hazard ratio estimates and 95% confidence intervals. Cancers in which TIS score is statistically significantly (*p* < 0.05) associated with good prognosis are highlighted in blue; significant associations with poor prognosis are in red. **b**-**e** Kaplan-Meier curves of overall survival split by TIS score tertiles within 4 selected tumor types: melanoma, sarcoma, pancreatic adenocarcinoma, and lower grade glioma

Since the TIS algorithm was developed in the context of single-arm studies of patients universally treated with single-agent pembrolizumab, its prognostic versus predictive value has yet to be established. In addition, considering that information about the nature, quantity, location, and functionality of immune infiltrates has been shown to contain prognostic information [3, 32–35], we explored the prognostic value of the TIS score in the absence of specific immune treatment. We performed univariate Cox regression predicting overall survival from TIS scores in each TCGA dataset. TIS scores were not statistically significantly prognostic in most cancers, with the notable exceptions of bladder cancer, cervical cancer, sarcomas, and melanoma, where a modest prognostic benefit (hazard ratio per unit of TIS score > 0.8) of high TIS score was observed; and in renal papillary cell carcinoma, lower grade glioma, and pancreatic adenocarcinoma, where TIS was associated with poor prognosis (hazard ratios > 1.2) Fig. 3. Of all these associations, only melanoma and lower grade glioma cancers had *p*-values corresponding to a False Discovery Rate [36] below 0.05. These findings can aid interpretation of single-arm studies comparing survival in high and low TIS score patients treated with an immunotherapy.

### TIS in breast cancer: relation to subtype, survival, and mutation burden

**Fig. 4:**
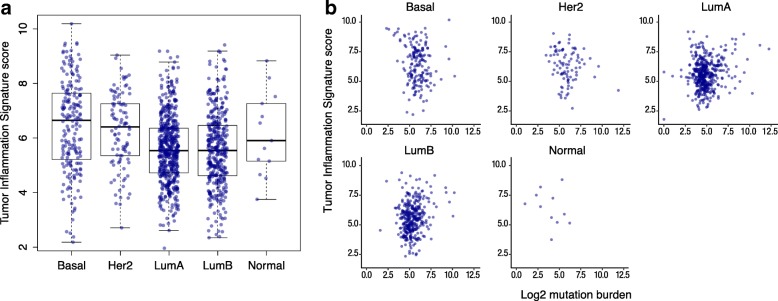
TIS scores across PAM50 subtypes. **a** Distribution of TIS score within each PAM50 subtype. **b** TIS plotted against log2 mutation load in each subtype

In order to explore the interaction between tumor-intrinsic genetic programs, tumor mutation load, and intratumoral immune response, we investigated the distribution of TIS scores and mutation burden within the intrinsic subtypes of breast cancers as defined by gene expression profiling, i.e., the PAM50 algorithm [37]. As shown in Fig. 4, TIS scores displayed considerable variability within all PAM50 subtypes. Average TIS scores were higher in the basal and Her2-enriched subtypes than in the luminal subtypes. However, between-subtype differences explain little of TIS’s variability in breast cancer: the variance between the subtypes’ means was 8% of the total variance of TIS score in the breast cancer samples.

Univariate Cox proportional hazard regression found no statistically significant association between TIS and overall survival in the TCGA breast cancer dataset. Cox models fit separately to each PAM50 subtype similarly showed no significant association. However, we observed that the subset of patients with the highest 10% of the TIS score range shows substantially improved prognosis (Additional file 9: Figure S5). This prognostic benefit of anti-tumor immunity is limited to the very highest TIS samples: the next highest 10% of TIS scores have prognosis equivalent to the lowest 80% of samples. These results echo those of Hendrickx et al. (2017) [38], who observed improved prognosis in breast cancers with the most favorable immune phenotype as measured by the immunologic constant of rejection (ICR). TIS scores and mutation load were minimally correlated within each PAM50 subtype Fig. 4, although Fig. 2 shows the weak correlation between mutation burden and TIS scores in breast cancer to be statistically significant.

### The TIS score reflects immune status rather than tumor-specific biology and is thus agnostic to tissue of origin

Tumors from different cells of origin tend to display highly divergent expression patterns, limiting the applicability of most gene expression algorithms across tumor types. In contrast, because the TIS depends primarily on genes expressed by immune cells or in response to immune signaling, it is plausible that its genes’ expression levels are driven by the magnitude of a tumor’s immune response and not by its cell of origin. To evaluate the applicability of the TIS algorithm across tumor types, we examined the extent to which the TIS genes’ expression levels depend on tumor type versus overall immune status as measured by TIS algorithm.

**Fig. 5:**
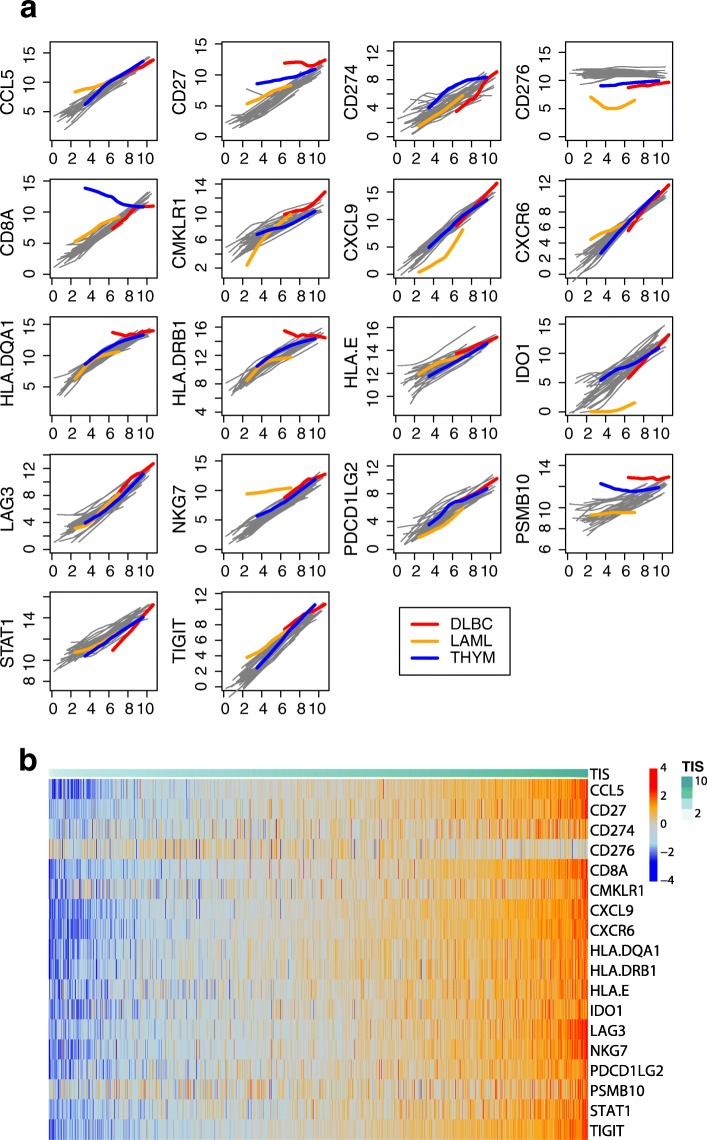
Algorithm genes depend more on TIS score than on cancer type. **a** Each gene is plotted against TIS score, with separate lowess lines fit for each cancer type. Immune-derived tumors are highlighted; other solid tumors are shown with grey lines. A gene with no dependency on tumor type would have the same association with TIS score in each cancer type, and the lines for each cancer type would be perfectly overlapping. A gene with problematic dependency on cancer type would have lines with markedly different slopes, intercepts, or shapes. **b** Samples are ordered from lowest to highest TIS score. The top color bar shows TIS score

Figure 5 shows each gene’s association with TIS score within each cancer type. Apart from the few exceptions described below, all algorithm genes increase with TIS score, and each gene’s lowess fit [39] to TIS score varies little between cancer types. This expression pattern is consistent with a model in which the algorithm genes measure immune-related transcriptomic activity and are minimally influenced by tumor type-specific expression. We generally do not observe expression patterns indicative of algorithm genes behaving differently across tumor types, for example a gene that is uniformly elevated or suppressed in a tumor type, or whose association with the TIS algorithm is different between cancer types.

There are exceptions to this pattern. First, the gene CD276, which codes for B7-H3, is uncorrelated with TIS score. Second, the 3 cancers in TCGA arising from “immune” cells, i.e., thymoma, acute myeloid leukemia (AML), and diffuse large B-cell lymphoma (DLBCL), all display expression patterns consistent with an effect of tumor type on the algorithm. In thymomas, CD8A expression, which has been shown to quantify CD8 T-cells in tumor samples [40], is high across all TIS scores and weakly negatively correlated with TIS score, likely because the tumor occurs in the thymus, the site of lymphocyte (T and B cell) maturation. In AML, the trends for CD276, IDO1, and NKG7 all have substantially different intercepts and slopes than seen in the other tumor types. In DLBCL, CD27, HLA-DQA1, HLA-DRB, and PSMB10 are all high across all levels of TIS scores. The uniformly high TIS scores in DLBCL likely result from tumor-intrinsic expression of algorithm genes rather than truly high anti-tumor immunity; Fig. 5 details the TIS genes that have idiosyncratic expression in DLBCL. Close analysis of the coordinate expression of TIS genes permits appropriate interpretation of clinical settings where the signature can confidently be deployed.

These results indicate that the TIS score interpretation as a measure of the adaptive immune response may be biased in cancers that affect cells of the immune system. However, the unremarkable expression patterns of the majority of TIS genes in these cancers suggest that an adapted TIS score with the “offending” genes removed could perform as it does in other cancers.

To visualize the results of Fig. 5 in another way, we created a heat map of the TIS algorithm genes in all of TCGA Fig. 5.

Additional file 4: Figure S2 quantifies the visual evidence of Fig. 5. Linear mixed models were used to estimate the variance in each gene’s expression attributable to tumor type. For most algorithm genes, the variance due to tumor type is a small proportion of total variance. The TIS normalization (housekeeping) genes show low variance within and across tumor types.

### Association between TIS scores and the transcriptome.

Biological processes that are negatively correlated with TIS scores could represent targets for future immunotherapies. In order to identify these processes, we searched the whole transcriptome for gene sets with persistent negative correlations with TIS. To do so, we evaluated the association between single gene expression levels and TIS scores in each cancer type using univariate linear regression. Genes with strong positive associations with TIS scores were far more common and more strongly correlated with TIS than genes with negative associations (Additional file 8: Figure S3), which is expected, because genes expressed by immune cells will tend to be correlated with the total level of adaptive immunity. Because genes that are persistently negatively associated with TIS may indicate alternative immune-inhibitory mechanisms, we used gene set analysis (GSA) [18], which summarizes the extent to which a gene set is positively or negatively associated with a condition, to search for GO terms with strong negative associations with TIS scores**.** Additional file 5: Figure S4 shows the 50 GO terms with the lowest GSA scores across all cancer types. Different tumor types have different GO terms associated with low TIS. The most frequent negatively-associated GO terms largely involve metabolism, which may reflect transcriptionally ‘lean’ tumors that have eluded immune detection as posited by Turan et al., (personal communication). Alternately, it may also reflect the suppressive effects of IFN signaling on cell growth that would lead to lower tumor cell metabolism in TIS-high tumors [41]. For each tumor type, Additional file 6: Table S1 lists the GO terms that are negatively associated with TIS scores, as defined by GSA scores < 1.

### Association between TIS scores and immune checkpoint genes

**Fig. 6:**
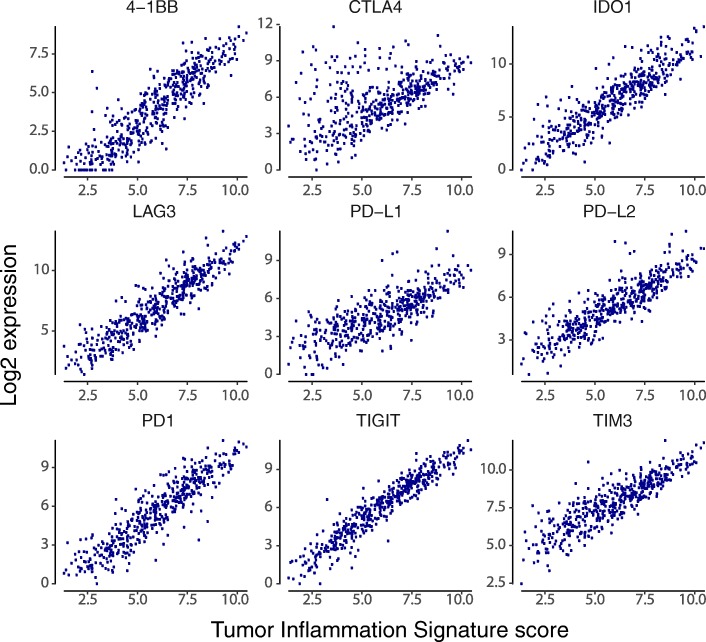
Expression of immunotherapy target molecules versus TIS in melanoma. Log2 expression of drug target genes is plotted against TIS scores in the TCGA melanoma dataset

We specifically explored the association between TIS scores and genes coding for immunotherapy target molecules. Many of these genes are in the TIS algorithm, including IDO1, LAG3, PD-L1 (CD274), PD-L2 (PDCD1LG2), and TIGIT; however, no single gene contributes enough to TIS to cause a spurious correlation between its expression and TIS score. Every immunotherapy target examined is positively correlated with TIS scores Fig. 6, suggesting that TIS scores could be of predictive value for all inhibitors of these targets. CLTA4 is the greatest departure from this trend, possibly reflecting the unique role CTLA4 plays in limiting the initial priming of T cells rather than suppressing T cell function after activation Fig. 6. Additional file 7: Figure S8-S39 show TIS score versus individual checkpoint genes in all tumor types. TIS score and PD-L1 are correlated, but not redundant: at any given TIS score, PDL1 (CD274) has an expression range of approximately 4 log2-units, or 16-fold on the linear scale. One potential explanation for this may be specific post-transcriptional regulation of immune checkpoint molecules, e.g., loss of miRNA binding sites via 3’ UTR deletion of the PD-L1 transcript 26].

**Fig. 7:**
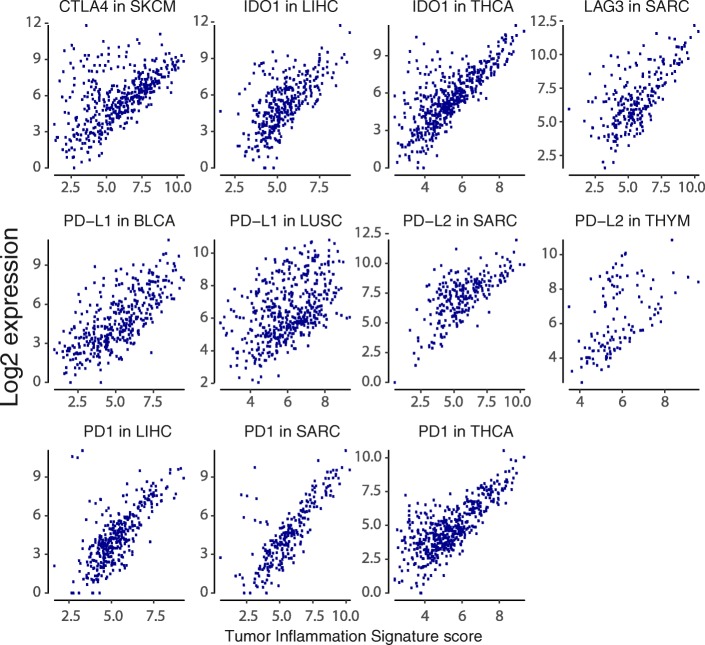
Instances of a subtype with high checkpoint expression but low TIS scores

Alongside the general trend for all checkpoint genes to be highly correlated with TIS scores in all cancers, there are cancer types in which a subset of patients has high expression of a checkpoint gene despite low TIS scores. Notable instances of this pattern are shown in Fig. 7.

## Discussion

It is now recognized that the immune system can be monitored and the results used to guide therapeutic decisions [42]. Using gene-expression profiling signatures, 2 major subsets of advanced solid tumors can be identified: those with a T cell inflamed tumor microenvironment, which have a signature of a pre-existing adaptive immune response, and non-T cell inflamed tumors, which lack evidence of a pre-existing adaptive immune response [43].

In the context of the current treatment landscape (i.e., checkpoint inhibitors) the tumor inflamed phenotype has been associated with response to these agents, e.g., [12, 33, 44, 45]; therefore, understanding whether a tumor has an inflamed or non-inflamed phenotype should be the starting point in the immunologic characterization of any tumor samples.

Currently there are no analytically and clinically validated gene expression-based tests for clinical use measuring tumor cell inflammation, although there have been previous efforts to characterize immune responses in tumors based on transcriptional profiling. Rooney et al. [15] use a set of genes associated with cytolytic activity to rank-order tumor types by immune response in a way that substantially agrees with our own approach. Two independent groups, Coppola et al. [16] and Bindea et al. [46] also use gene expression profiling to characterize immune response within colorectal cancer, but these patterns of gene expression have not been extended beyond CRC and have not been associated with response to immunotherapy.

The TIS is an IUO 18-gene signature that detects an adaptive immune response within tumors by measuring expression of genes associated with cytotoxic cells, antigen presentation, and interferon gamma (IFNγ) activity [47]. The TIS has previously been shown to enrich for a population of patients who respond to the anti-PD1 agent pembrolizumab and has been analytically validated as a clinical trial assay for investigational use only and has been tested retrospectively in clinical trials [12, 47]. Since the genes in the signature measure immune-intrinsic transcriptomic activity with minimal contribution of tumor-intrinsic gene expression, the signature may prove to be pan-cancer or tumor-type agnostic. In the present study we applied the TIS across a range of tumor gene expression data downloaded from the TCGA in order to characterize the immune profile of a wide range of immunotherapy-naïve tumor types.

As expected, we found that tumors with greater sensitivity to anti-PD-1 blockade tend to have higher average TIS scores. The tumor ranking we observed is comparable with that obtained by Spranger et al. (2016) [48], who interrogated TCGA across 30 solid tumor types with a 160-gene expression signature for T-cell inflammation. In that work, a wide range of abundance of the T cell-inflamed tumor microenvironment gene signature was observed both within and across tumor types, with the highest fraction seen in clear cell kidney cancer and lung adenocarcinoma. Our work generated similar results; however, since the genes in the Spranger signature have not been published, direct comparison is not possible. Furthermore, since the TIS (reagents, instrumentation, algorithm and software) has been analytically developed as an IUO diagnostic assay, it may be prospectively deployed for patient selection in clinical trials.

Notably, TIS genes have highly conserved co-expression patterns across tumor types, consistent with a model in which the genes measure immune phenotype independent of the tumor cell of origin. In addition, while TIS scores are higher in classically immunogenic tumor types, they display a significant amount of intersample variability within most tumor types, and a subset of patients can be identified who possess elevated TIS scores, consistent with responsiveness to anti-PD-1 blockade, in all tumor types but with different prevalence. These findings raise the possibility that TIS, much like MSI/dMMR, could be used as pan-tumor biomarker enriching for patients likely to respond to single agent anti-PD-1/PD-L1 treatment. Furthermore, by simultaneously evaluating expression of other immune checkpoints relative to TIS expression, it may be possible to identify tumors with high checkpoint expression/low TIS score that are candidates for responding to the “right” checkpoint inhibitor despite being “immune cold”, as measured by TIS score.

Mutation load has been shown in retrospective analysis to be a predictive biomarker for clinical benefit from single-agent anti-PD-1/PD-L1 agents in certain tumor types such as melanoma, NSCLC, bladder, and HNSCC [9, 49–51]. Similarly, TIS’s association with clinical benefit (i.e. reduction in tumor burden) from the same agents has been shown in many of the same tumor types [12, 51–53]. Notably, our study found only weak correlation between TIS scores and mutation load; although this is consistent with previous observations from others [48, 54], it also suggests that the 2 biomarkers are not fully overlapping and might contribute orthogonal information in certain tumor settings [55]. Specifically, total mutation load is a surrogate measure of the intrinsic potential tumor antigenicity (but it is “upstream” of the immune response cascade), while TIS score is a direct measure of the ongoing immune response within the tumor, but is sensitive to sampling bias due to the heterogeneity of the tumor microenvironment. A combination of the 2 biomarkers could therefore increase accuracy in identifying patients who can potentially benefit from checkpoint inhibitors.

In many cases, for example in the 3 kidney cancer types in TCGA, ranking tumor types by median TIS score showed superior association to reported clinical sensitivity to PD-1/PD-L1 blockade than ranking of the same tumors by mutation load; thus, in tumor types with limited variability of mutation burden, TIS may have more predictive power than other profiling techniques. For instance, in head and neck tumors, some cases are caused by smoking and will have high mutation loads, but some cases are caused by human papillomavirus (HPV) and would have immune responses directed against the viral antigens. As shown by Haddad and colleagues (2017) [51], TIS predictive value in the context of single agent pembrolizumab is independent from HPV infection status while mutation load is only predictive in HPV negative tumors. Similarly, cervical tumors are also associated with HPV. These tumors would be expected to have high TIS and low mutation load, and this can be seen in the data (CESC and HNSC, Fig. 2).

TIS appears to be minimally prognostic in most, but not all cancers, perhaps due to the immune evasion strategies that must be established in order for the tumor to grow to the size that it is clinically detectable. This finding supports interpretation of TIS in single-arm clinical trials: if a survival benefit is seen in high TIS patients, it likely results from improved response to the drug and not to an inherent survival benefit of high TIS. In an important exception, this assumption does not apply in melanoma, where TIS was highly statistically significantly prognostic. TIS was also positively prognostic in bladder carcinomas, cervical carcinomas, and sarcomas. In these cancers, trials in high-TIS patients will suffer attenuated power if the placebo arm displays better survival than historical data [56]. The tumor types in which TIS score is negatively prognostic (kidney papillary, pancreatic, glioma) are known to be resistant to PD-1 therapy, and the prevalence of TIS positivity in these tumors is low. In contrast to the limited prognostic power of TIS, Ayers et al. reported that TIS was associated with objective response to pembrolizumab across a variety of tumors, so TIS has potential pan-cancer applicability in predicting response to PD-1 checkpoint blockade [12].

Finally, to screen for novel suppressive mechanisms, this study searched for biological pathways associated with low TIS scores. Metabolism, ribosomal, and telomere-associated pathways all predicted lower TIS scores in multiple cancer types. No targetable oncogenic pathways were associated with TIS.

This study has several technical limitations. The TIS algorithm is an investigational assay on the NanoString nCounter platform that was developed with data from clinical trials of the checkpoint inhibitor pembrolizumab, whereas TCGA samples were profiled using RNAseq from biopsies taken at diagnosis. Data from the nCounter-based assay differs from TCGA RNASeq data in several ways: in the NanoString assay, a different platform is used to measure gene expression, patient sample data are normalized to an in-vitro transcribed RNA reference sample to control for technical effects, FFPE tissue is used, and tissues with < 50% tumor are macrodissected. To the extent that these differences influence gene expression measurements by changing probe efficiencies, a reasonable model for most of the effects, TCGA TIS scores will only be shifted by a constant from what the TIS assay would have returned from the same tissues. The effect of different macrodissection protocols, however, is variable and hard to predict, and could plausibly cause more complex inter-assay TIS score differences.

Another limitation of the current study is that it relies upon the early stage tumors that were collected in the TCGA cohort and thus may not be well matched to the late stage disease where immunotherapy is being applied clinically today. However, at the time of this work, large publicly available gene expression profiling datasets of late stage cancer cohorts are not available. Furthermore, clinical trials in immunotherapy are being deployed earlier in the course of disease where the TCGA cohort is more representative of the clinical population.

Despite these limitations, this analysis of TCGA data shows the value (both from a mechanistic and a clinical point of view) of analytically validated gene expression signature measuring the level of T cell inflammation as an immune-phenotyping tool across different histologically defined tumor types. It also highlights the need for broader characterization of the mechanisms of immune evasions operating within the T cell inflamed and non-inflamed tumors, ideally in the same assay, maximizing the clinically actionable information extractable from a single tumor sample.

## Additional files

Additional file 1: **Figure S7.** Distribution of TIS scores in stage IV disease. TIS scores are shown for all TCGA patients with stage 4 disease. Cancer types are ordered by median TIS score in all patients, identical to Fig. 1. (PDF 30 kb)

Additional file 2: **Figure S1.** Meta-analysis of TIS score with objective response rates. [21, 50, 57–106]. (PDF 6 kb)

Additional file 3: **Figure S6.** Distribution of TIS scores within two MSI status categories and three cancer types. Points show individual samples’ TIS scores. (PDF 31 kb)

Additional file 4: **Figure S2.** In order to assess whether/how cancer cell of origin could directly affect the expression of TIS genes, the observed expression level for each gene versus the expected expression level based on total TIS score was evaluated. Specifically, for each algorithm gene, a linear mixed model (LMM) was fit predicting the gene’s log2 expression from TIS score and cancer type, with cancer type modelled as a random effect. The LMM’s variance term for cancer type was compared to each gene’s marginal variance across TCGA datasets. (PDF 4 kb)

Additional file 5: **Figure S4.** GO terms with the strongest negative association with TIS. Color shows GSA score. (PDF 16 kb)

Additional file 6: **Table S1.** For each cancer type, GO terms with negative associations with TIS, defined as a GSA score below − 1. (CSV 48 kb)

Additional file 7: **Figures. S8–S39.** Checkpoints versus TIS score in all tumor types. (PDF 701 kb)

Additional file 8: **Figure S3.** Volcano plot showing association between single genes and TIS in TCGA SKCM. (PDF 1092 kb)

Additional file 9: **Figure S5.** Association between TIS score and breast cancer survival. Breast cancers were divided in 4 subsets based on their TIS scores. Kaplan-Meier curves and confidence intervals are shown for each subset. (PDF 17 kb)
