# Supplementary figures and images for: Pan-cancer adaptive immune resistance as defined by the Tumor Inflammation Signature (TIS): results from The Cancer Genome Atlas (TCGA)
Source: J Immunother Cancer. 2018 Jun 22;6:63. doi: 10.1186/s40425-018-0367-1 (PMC6013904; doi:10.1186/s40425-018-0367-1)

Tumor Inflammation Signature score

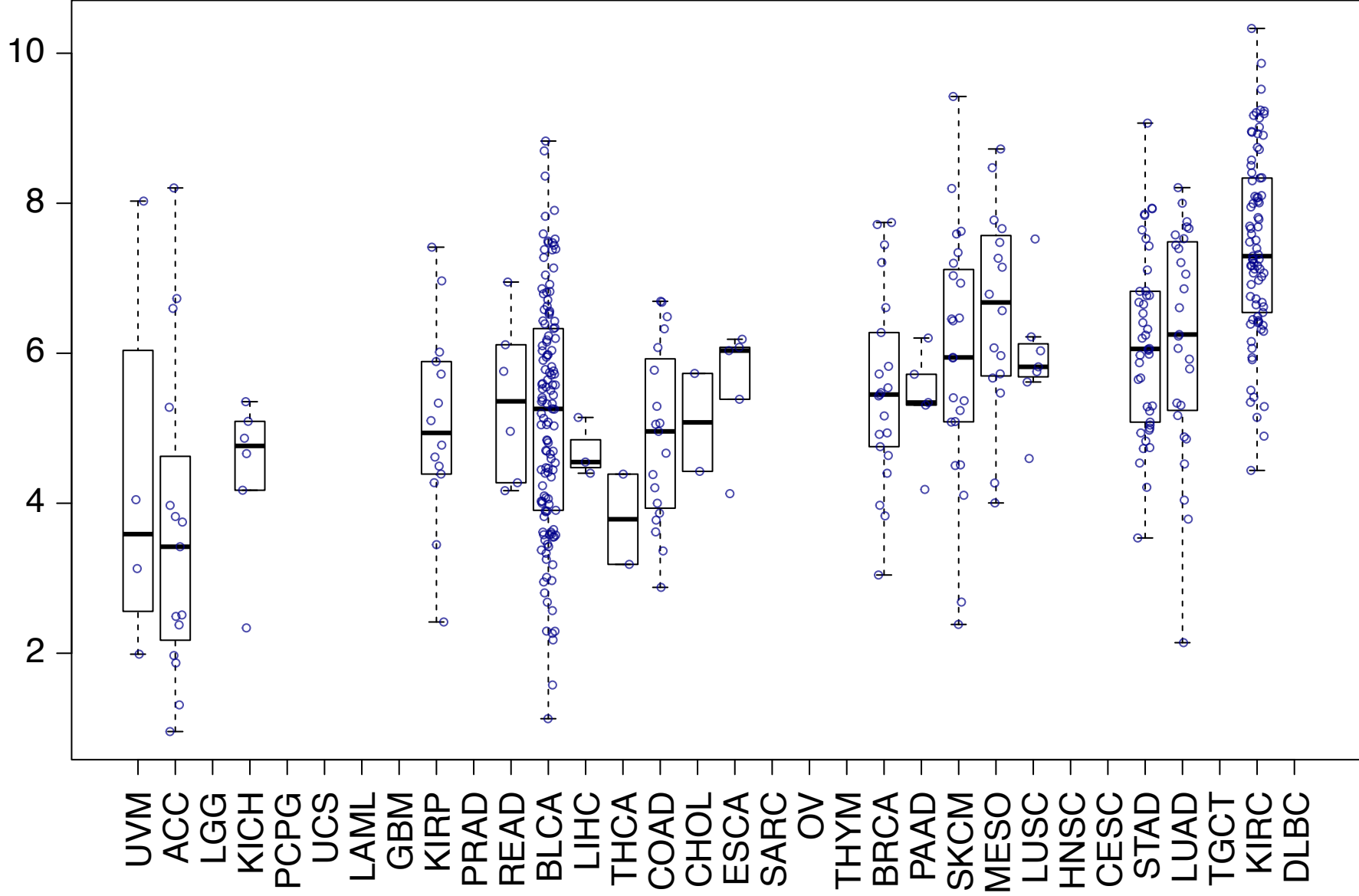

Supplement: Supplementary file 1 — Figure S7. Distribution of TIS scores in stage IV disease. TIS scores are shown for all TCGA patients with stage 4 disease. Cancer types are ordered by median TIS score in all patients, identical to Fig. 1. (PDF 30 kb) [file 40425_2018_367_MOESM1_ESM.pdf]

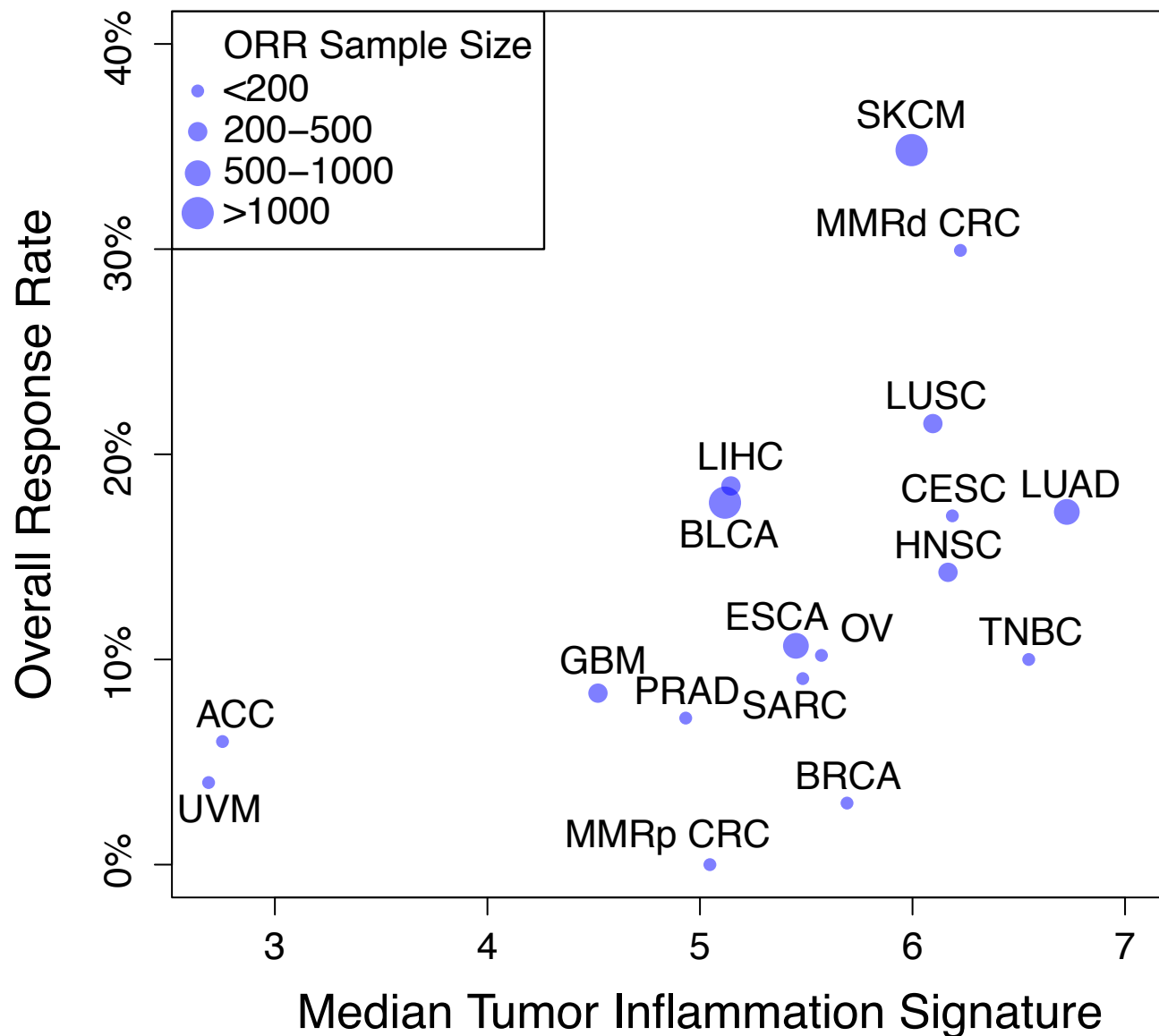

Supplement: Supplementary file 2 — Figure S1. Meta-analysis of TIS score with objective response rates. [21, 50, 57–106]. (PDF 6 kb) [file 40425_2018_367_MOESM2_ESM.pdf]

Tumor Inflammation Signature score

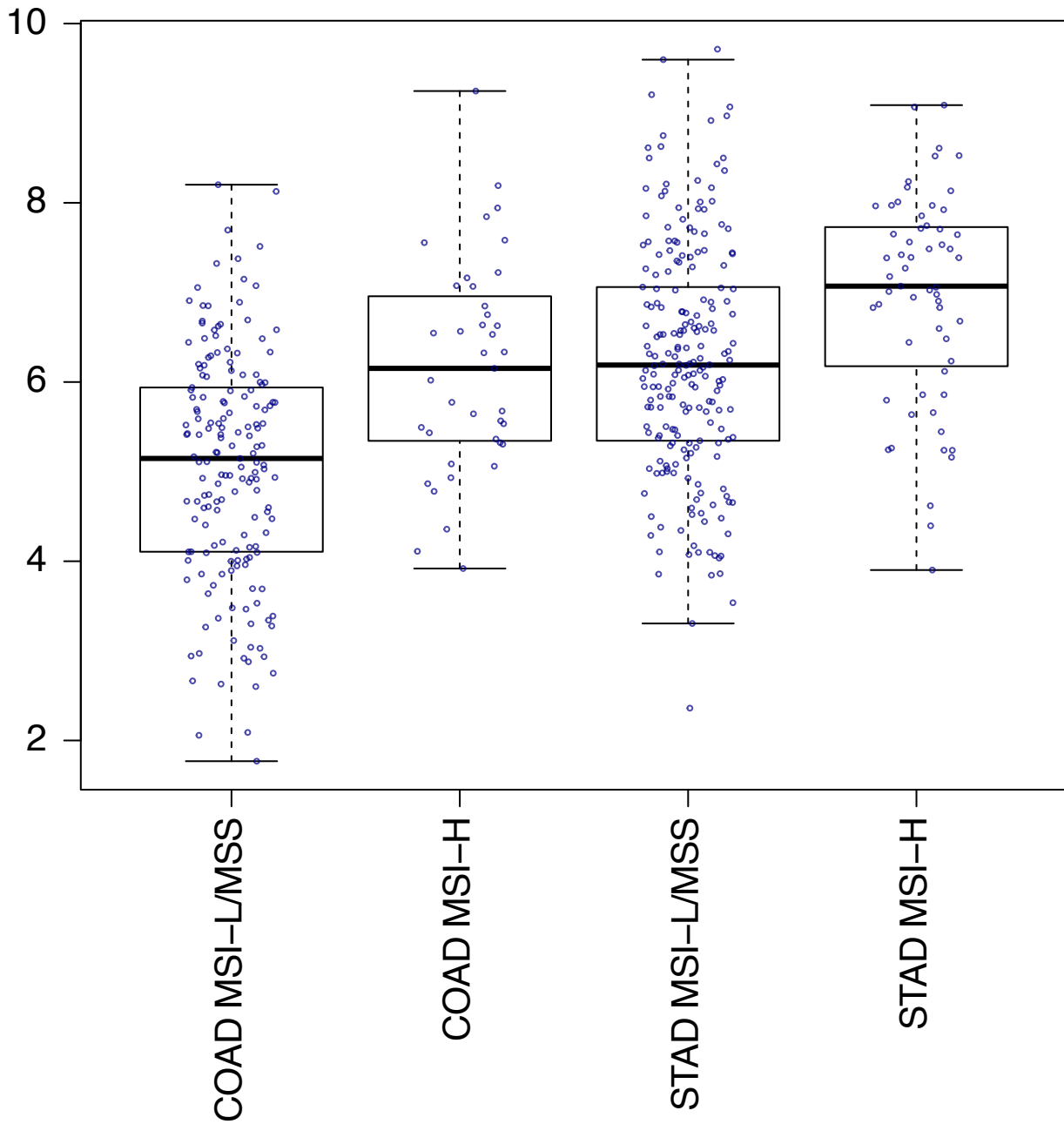

Supplement: Supplementary file 3 — Figure S6. Distribution of TIS scores within two MSI status categories and three cancer types. Points show individual samples’ TIS scores. (PDF 31 kb) [file 40425_2018_367_MOESM3_ESM.pdf]

Variance attributable  
to cancer type

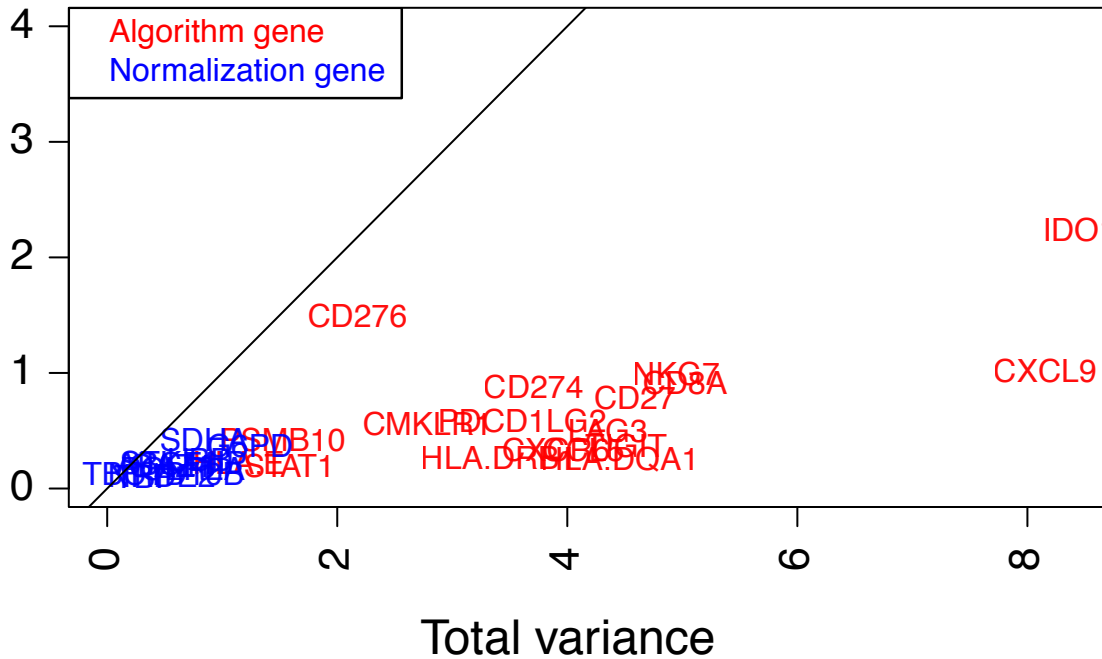

Supplement: Supplementary file 4 — Figure S2. In order to assess whether/how cancer cell of origin could directly affect the expression of TIS genes, the observed expression level for each gene versus the expected expression level based on total TIS score was evaluated. Specifically, for each algorithm gene, a linear mixed model (LMM) was fit predicting the gene’s log2 expression from TIS score and cancer type, with cancer type modelled as a random effect. The LMM’s variance term for cancer type was compared to each gene’s marginal variance across TCGA datasets. (PDF 4 kb) [file 40425_2018_367_MOESM4_ESM.pdf]

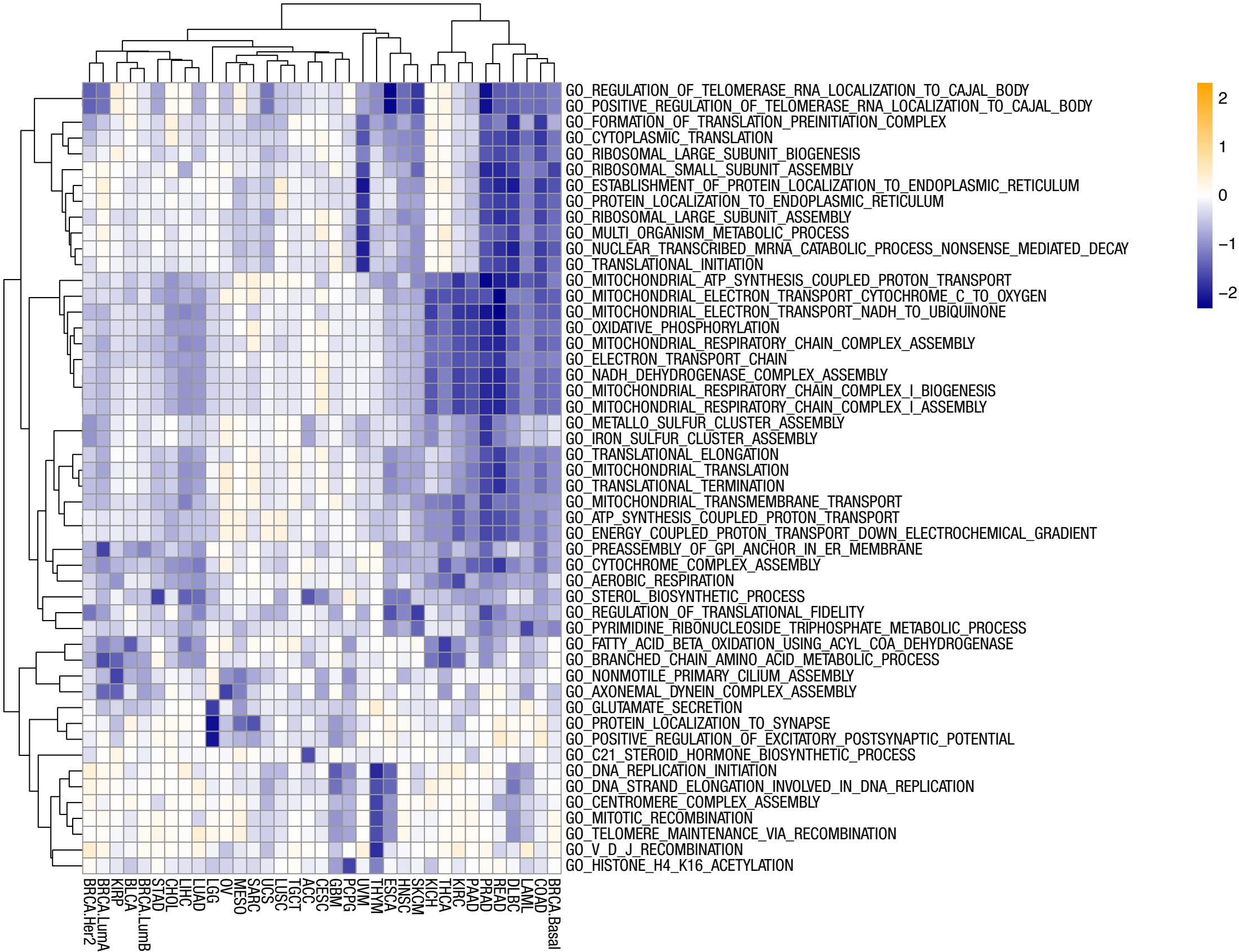

Supplement: Supplementary file 5 — Figure S4. GO terms with the strongest negative association with TIS. Color shows GSA score. (PDF 16 kb) [file 40425_2018_367_MOESM5_ESM.pdf]

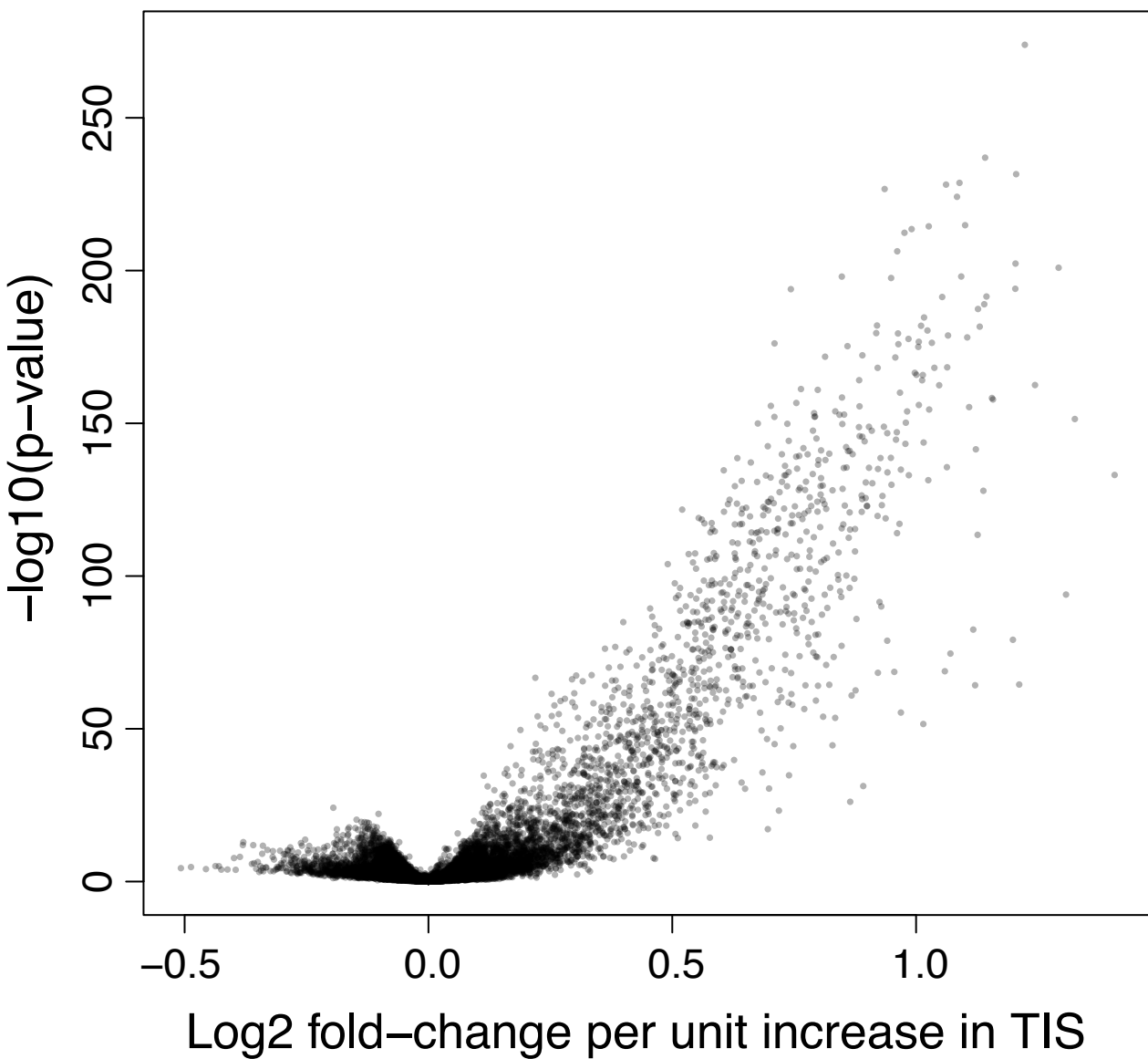

Supplement: Supplementary file 8 — Figure S3. Volcano plot showing association between single genes and TIS in TCGA SKCM. (PDF 1092 kb) [file 40425_2018_367_MOESM8_ESM.pdf]

TIS percentile    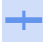 lowest 50    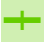 50–80    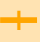 80–90    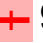 90–100

Survival probability

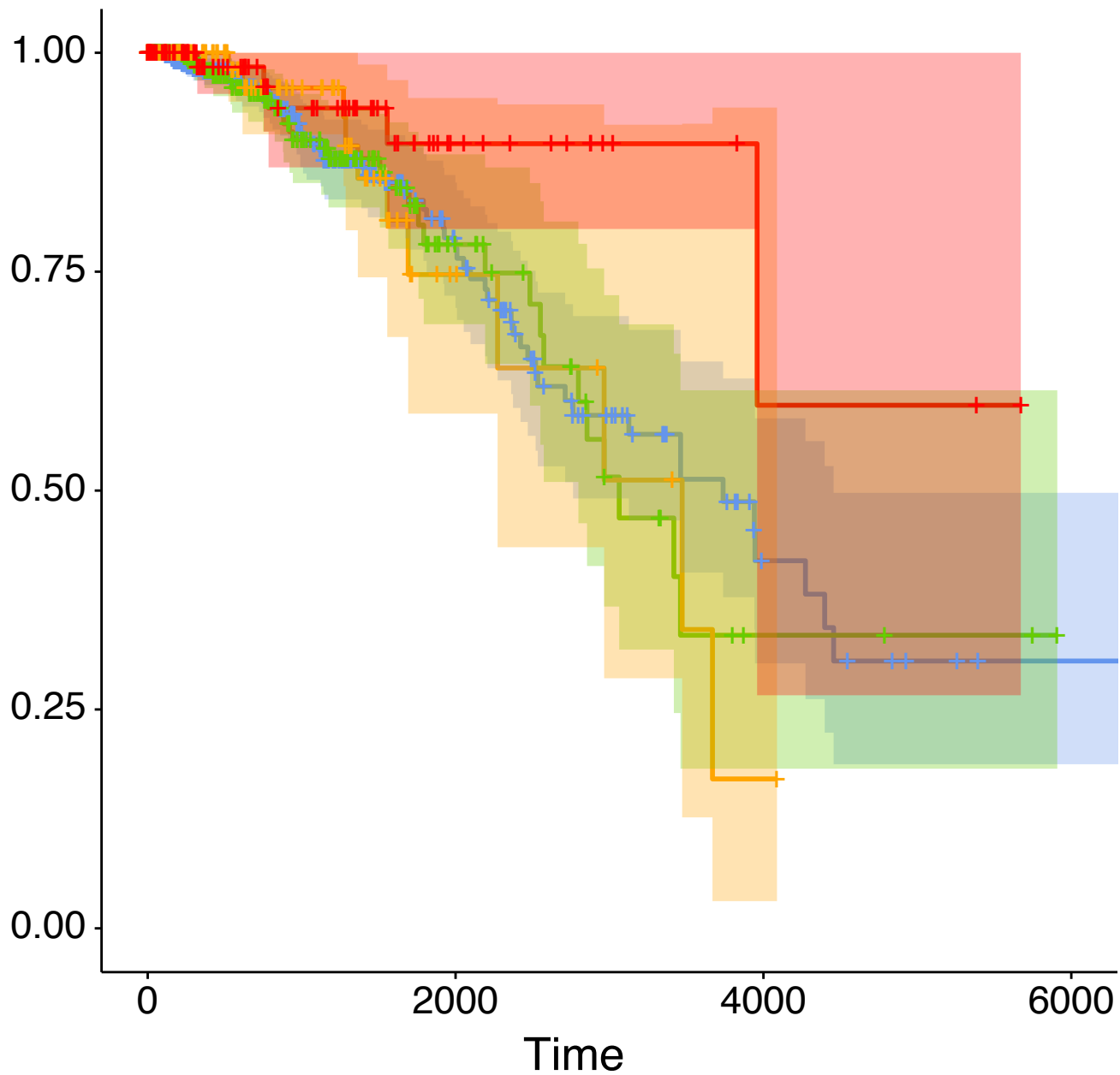

Supplement: Supplementary file 9 — Figure S5. Association between TIS score and breast cancer survival. Breast cancers were divided in 4 subsets based on their TIS scores. Kaplan-Meier curves and confidence intervals are shown for each subset. (PDF 17 kb) [file 40425_2018_367_MOESM9_ESM.pdf]
