# Supplementary material for: Pan-cancer adaptive immune resistance as defined by the Tumor Inflammation Signature (TIS): results from The Cancer Genome Atlas (TCGA)
Source: J Immunother Cancer. 2018 Jun 22;6:63. doi: 10.1186/s40425-018-0367-1 (PMC6013904; doi:10.1186/s40425-018-0367-1)
Supplement: Supplementary file 7 — Figures. S8–S39. Checkpoints versus TIS score in all tumor types. (PDF 701 kb) [file 40425_2018_367_MOESM7_ESM.pdf]

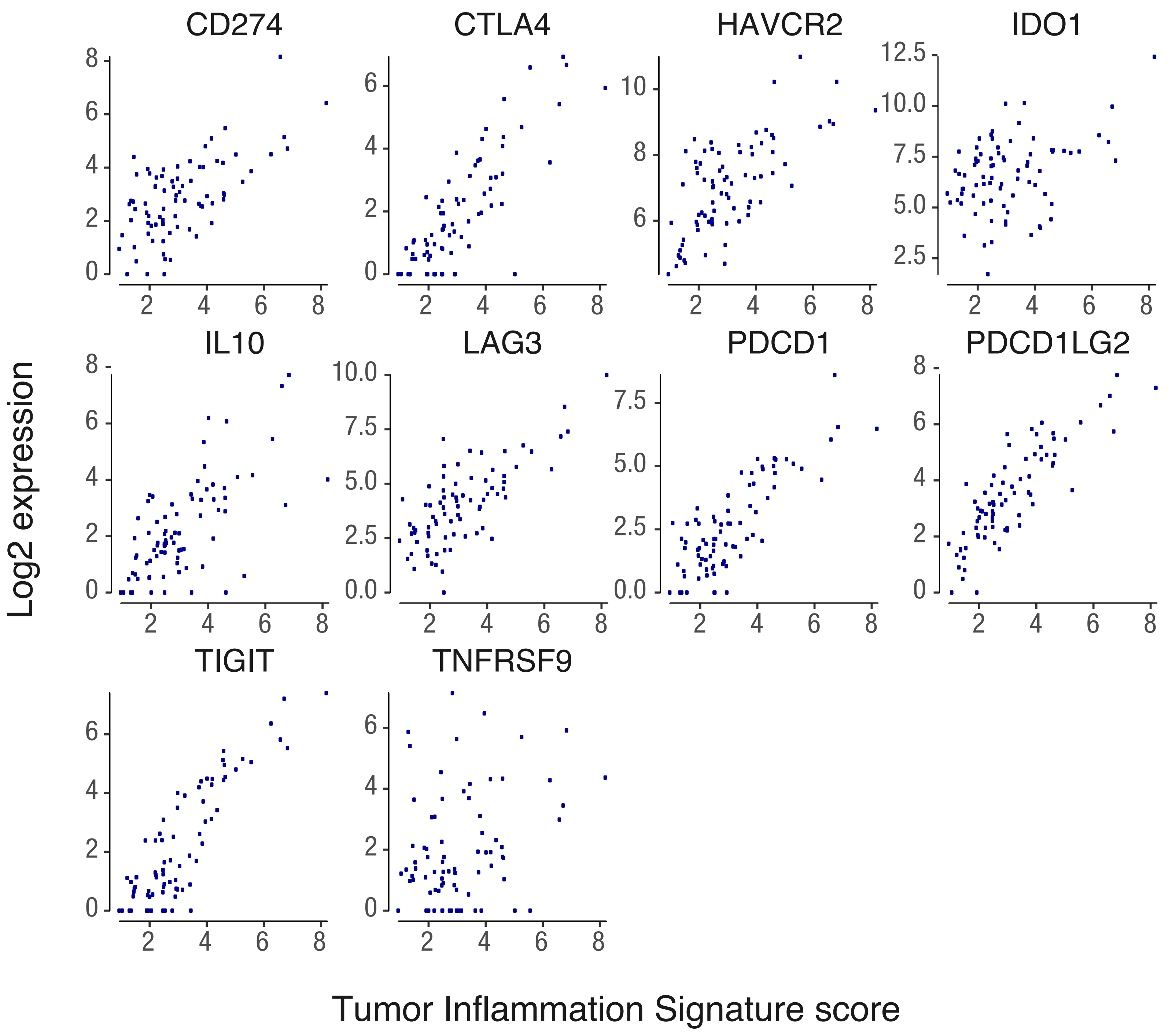
CD274
CTLA4
HAVCR2
IDO1
IL10
LAG3
PDCD1
PDCD1LG2
TIGIT
TNFRSF9
Log2 expression
Tumor Inflammation Signature score

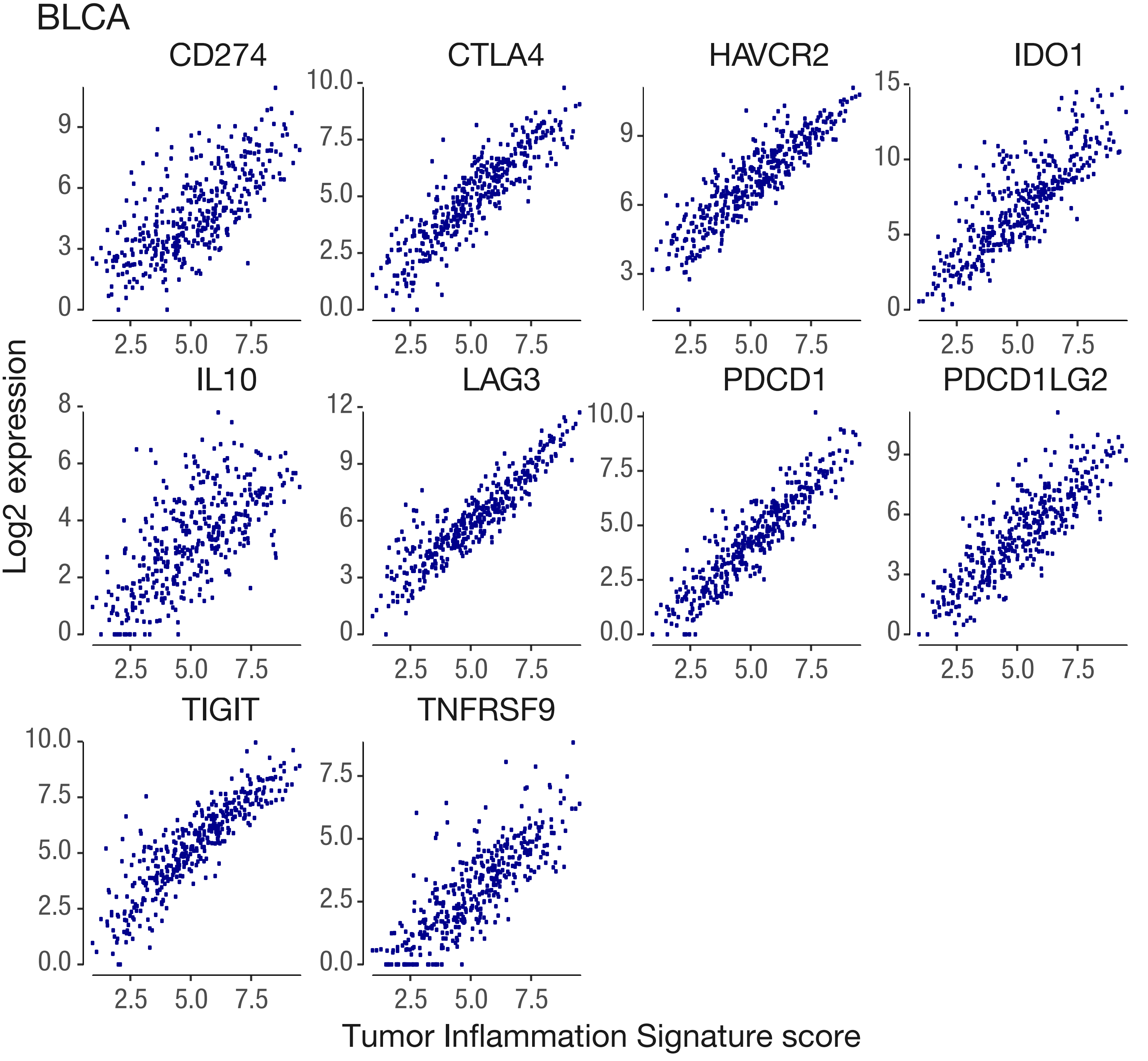
BLCA
CD274
CTLA4
HAVCR2
IDO1
IL10
LAG3
PDCD1
PDCD1LG2
TIGIT
TNFRSF9
Log2 expression
Tumor Inflammation Signature score

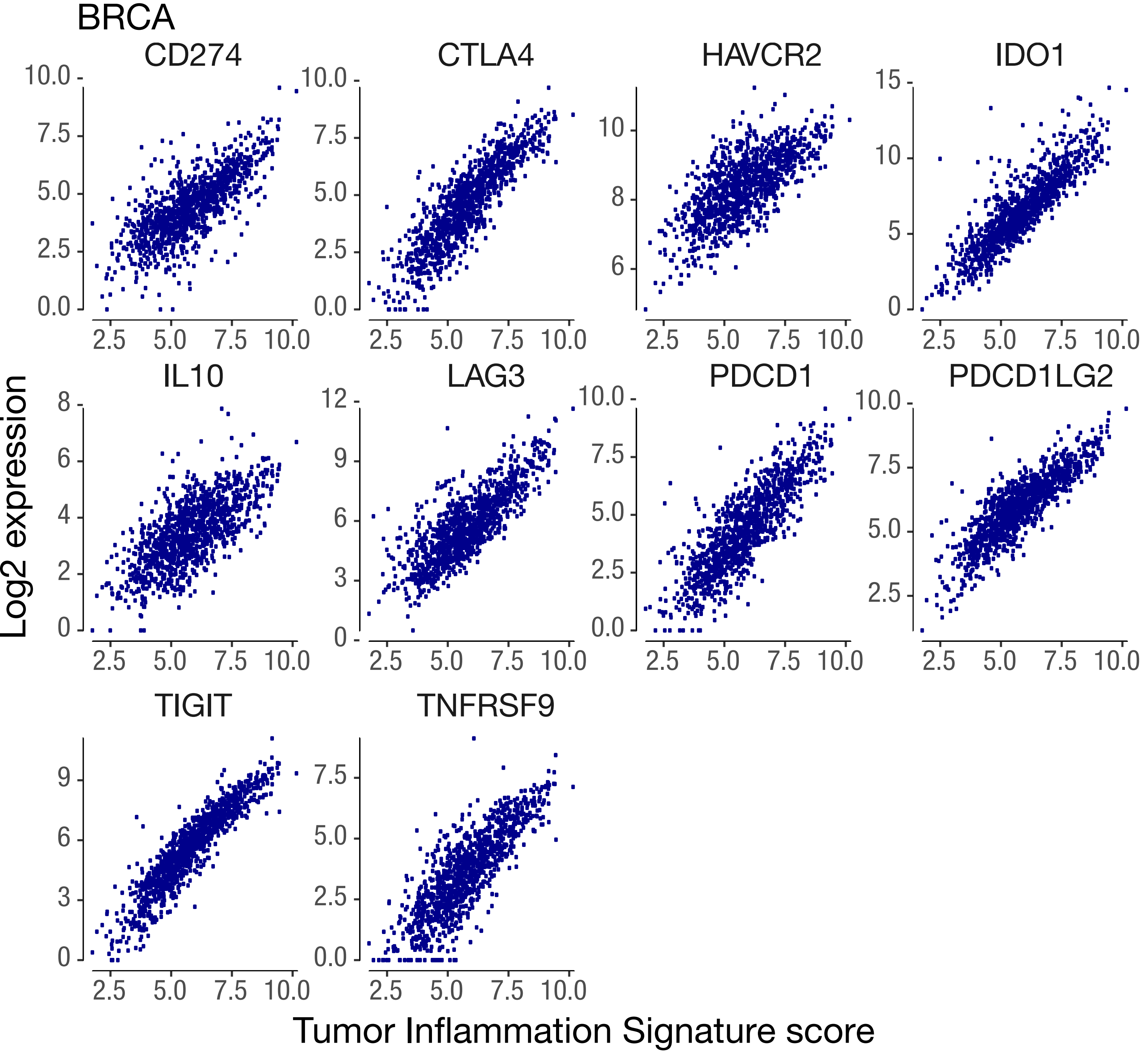
BRCA
CD274
CTLA4
HAVCR2
IDO1
IL10
LAG3
PDCD1
PDCD1LG2
TIGIT
TNFRSF9
Log2 expression
Tumor Inflammation Signature score

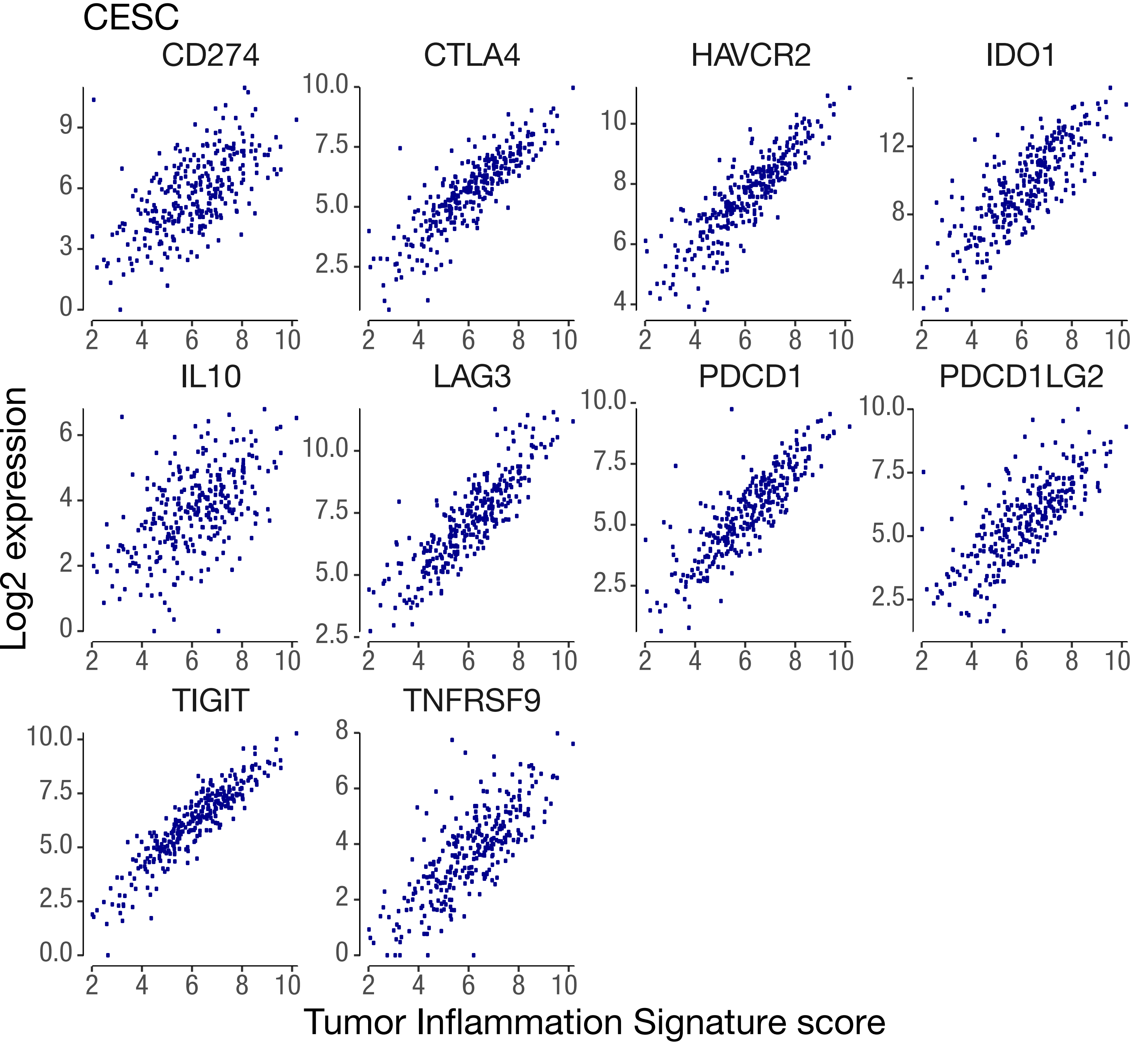
CESC
CD274
CTLA4
HAVCR2
IDO1
IL10
LAG3
PDCD1
PDCD1LG2
TIGIT
TNFRSF9
Log2 expression
Tumor Inflammation Signature score

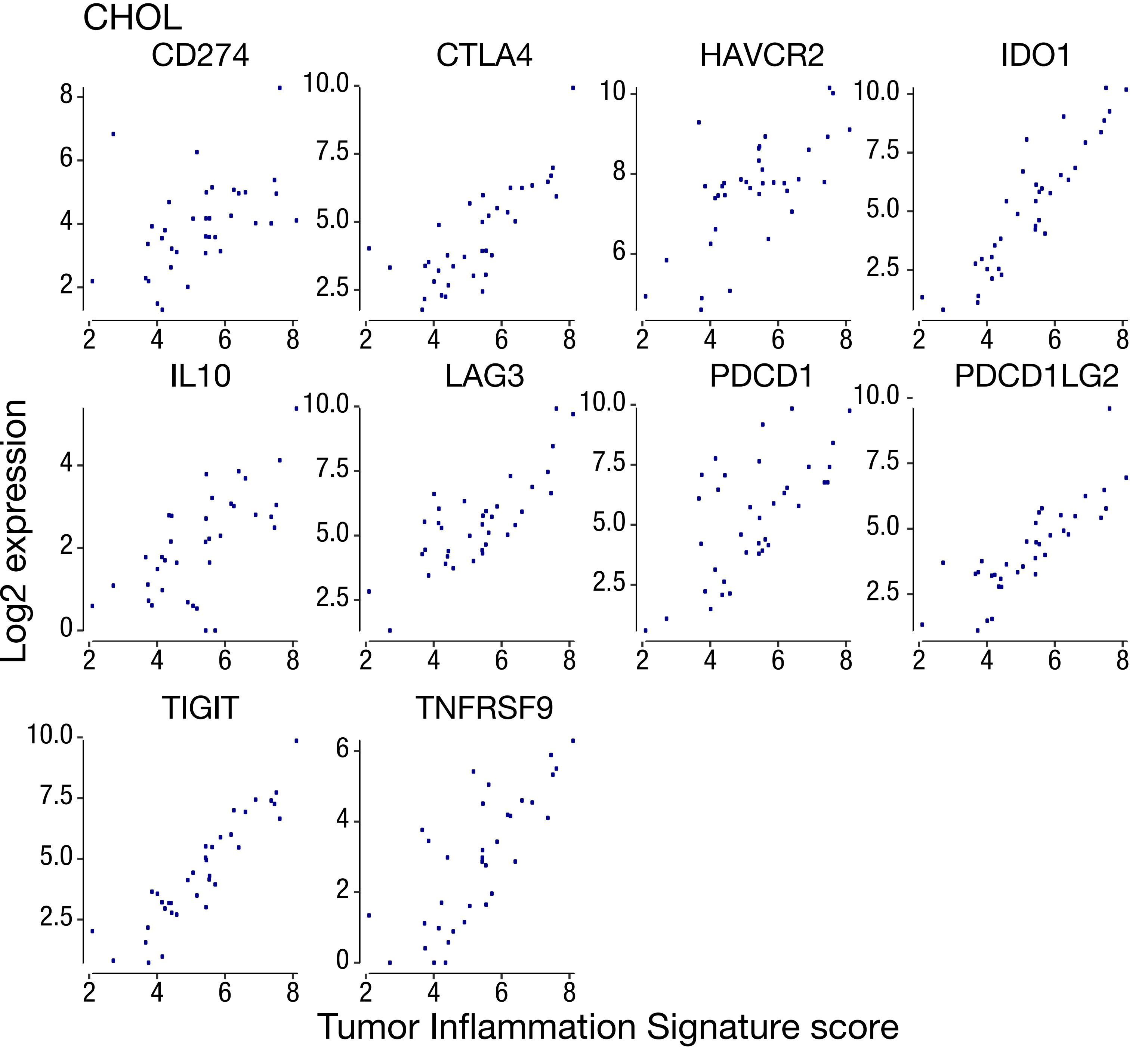

CHOL
CD274
CTLA4
HAVCR2
IDO1
IL10
LAG3
PDCD1
PDCD1LG2
TIGIT
TNFRSF9
Log2 expression
Tumor Inflammation Signature score

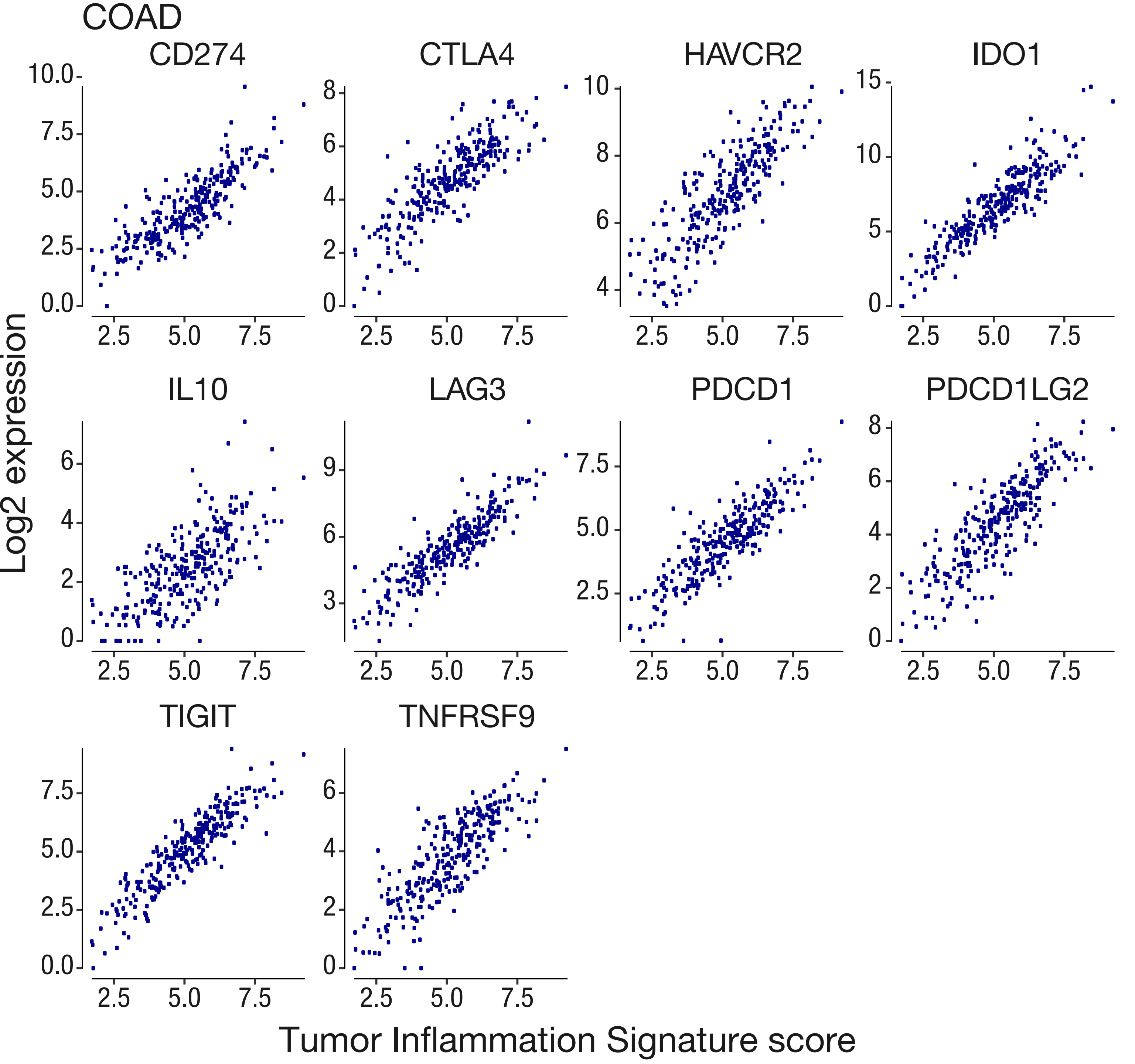

COAD
CD274
CTLA4
HAVCR2
IDO1
IL10
LAG3
PDCD1
PDCD1LG2
TIGIT
TNFRSF9
Log2 expression
Tumor Inflammation Signature score

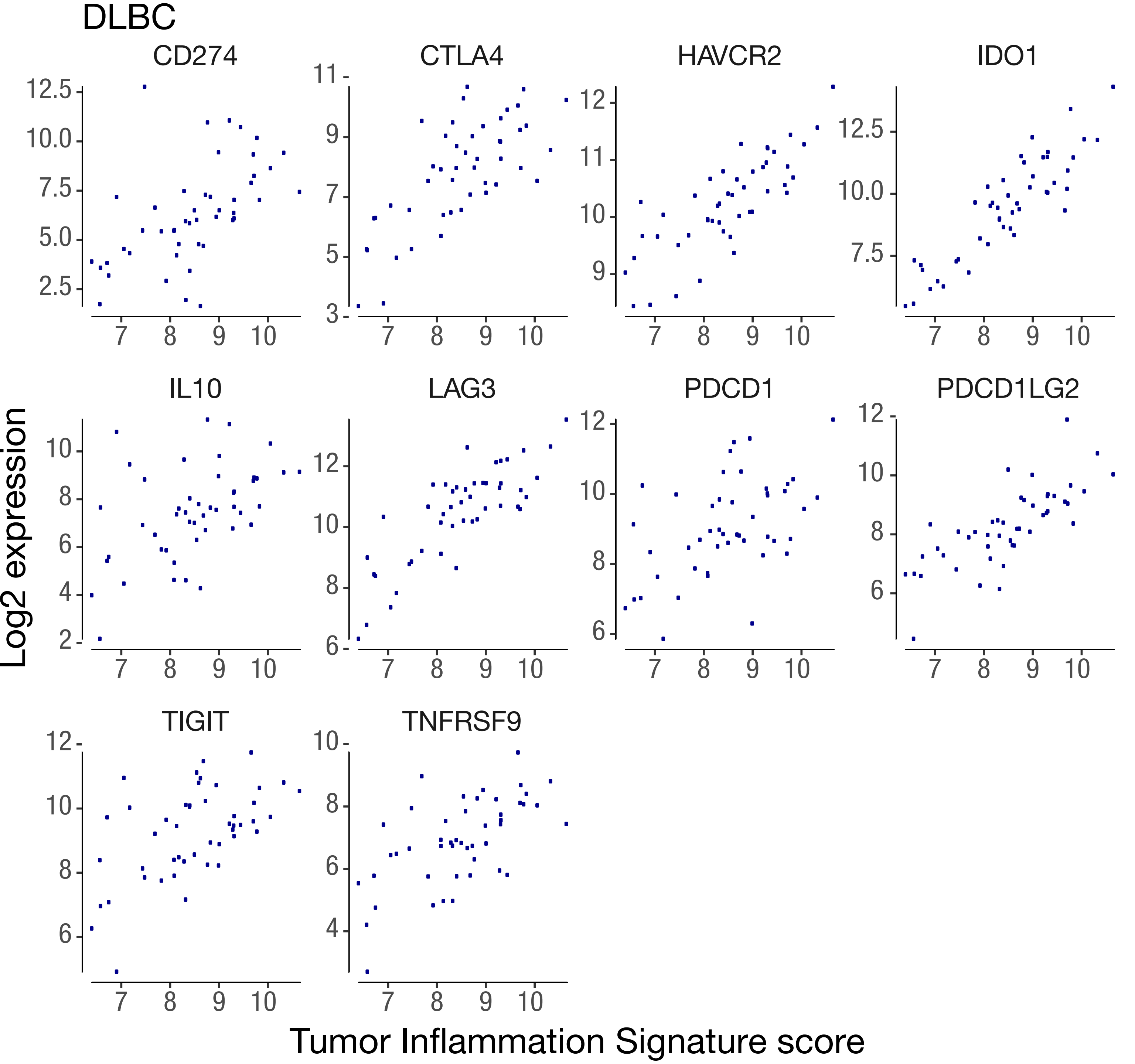
DLBC
CD274
CTLA4
HAVCR2
IDO1
IL10
LAG3
PDCD1
PDCD1LG2
TIGIT
TNFRSF9
Log2 expression
Tumor Inflammation Signature score

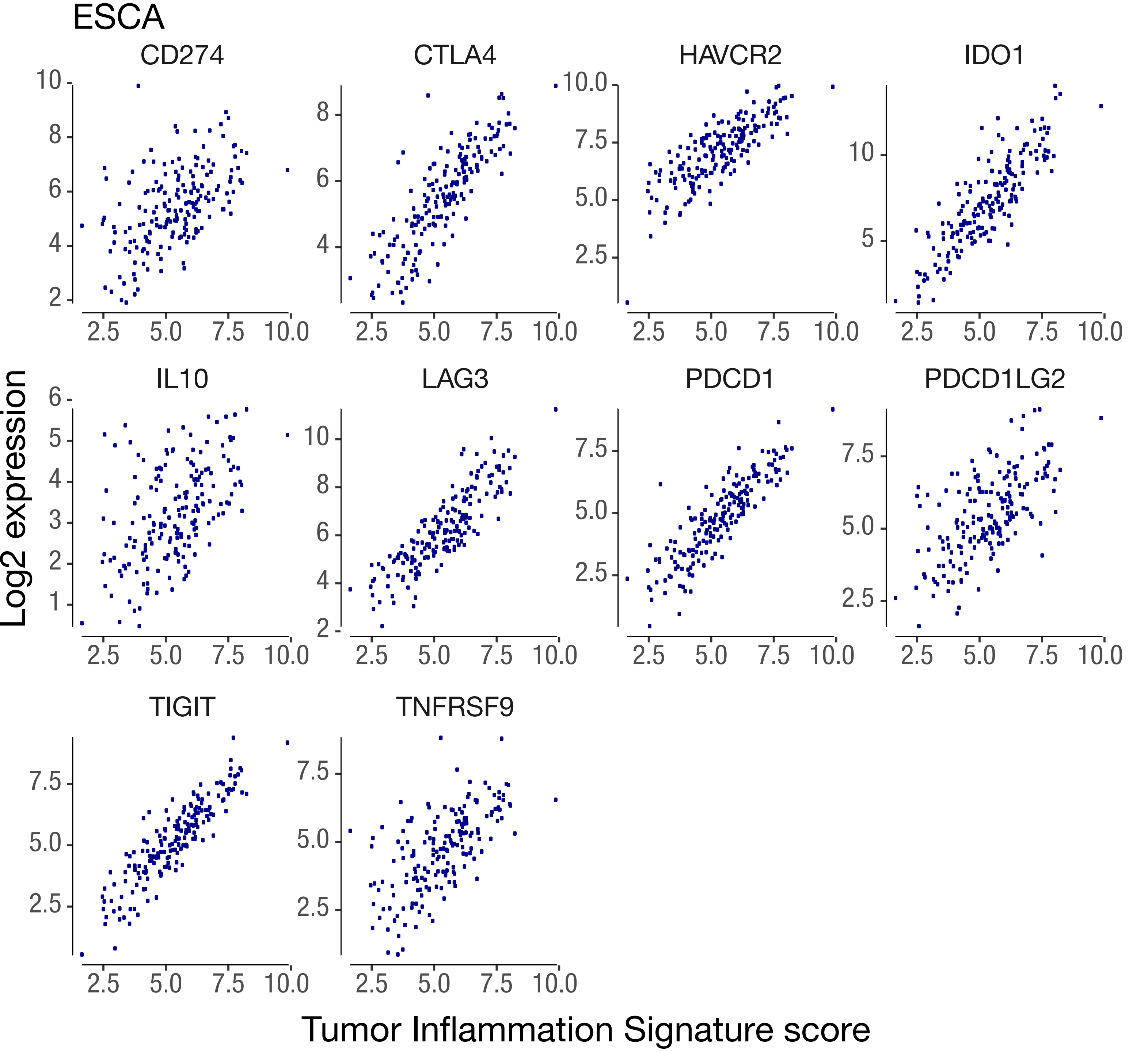
ESCA
CD274
CTLA4
HAVCR2
IDO1
IL10
LAG3
PDCD1
PDCD1LG2
TIGIT
TNFRSF9
Log2 expression
Tumor Inflammation Signature score

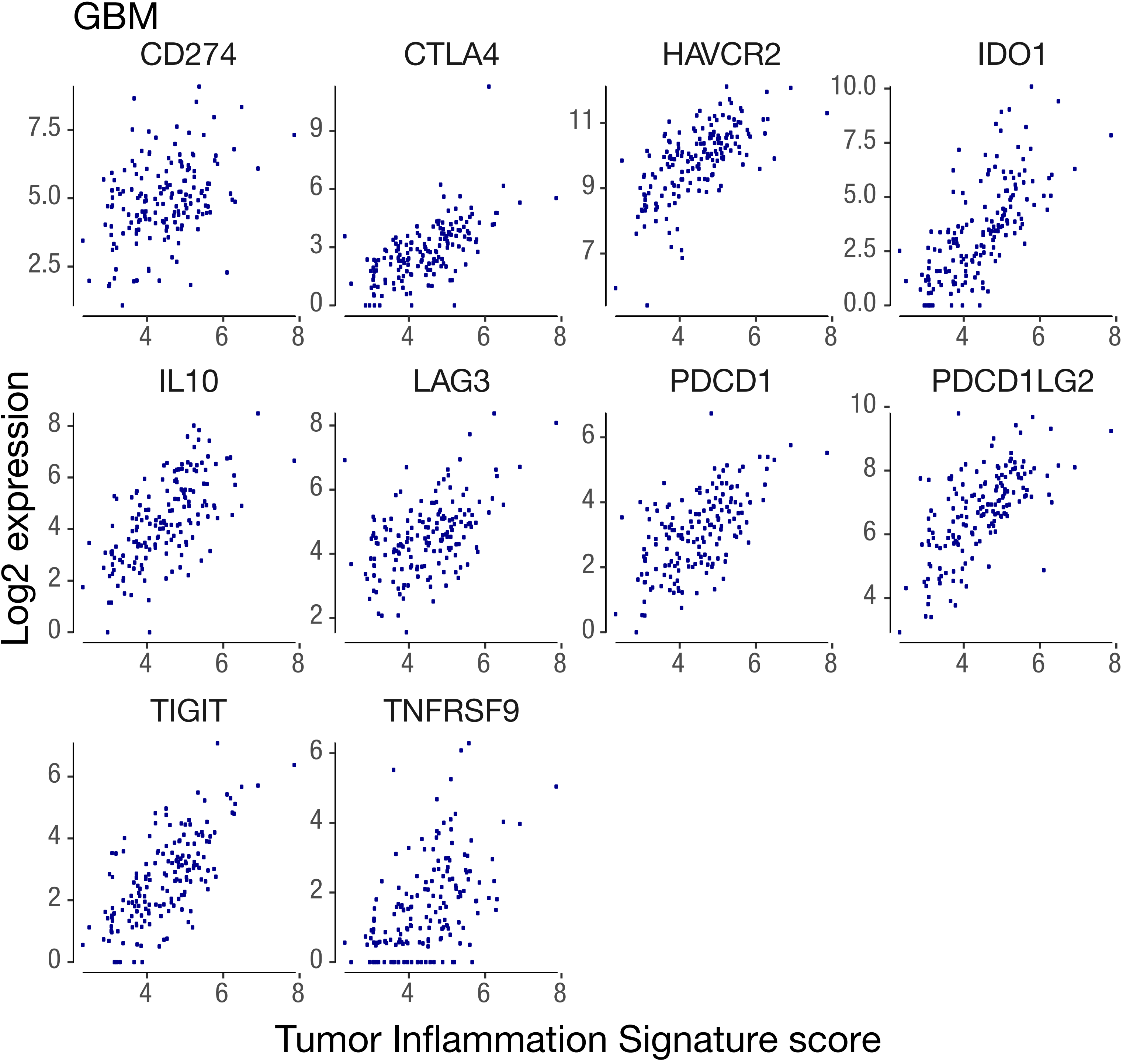
GBM
CD274
CTLA4
HAVCR2
IDO1
IL10
LAG3
PDCD1
PDCD1LG2
TIGIT
TNFRSF9
Log2 expression
Tumor Inflammation Signature score

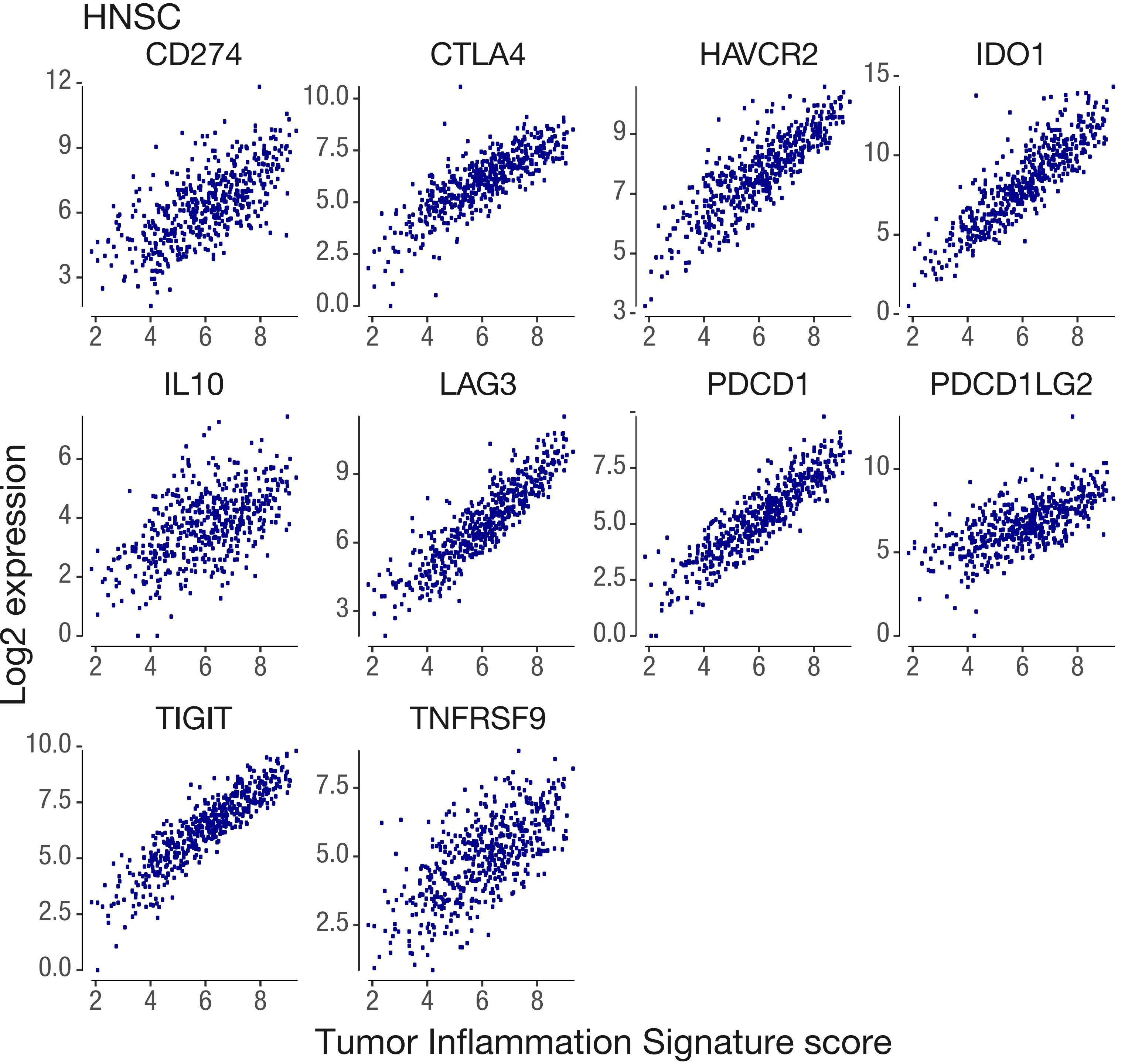

HNSC
CD274
CTLA4
HAVCR2
IDO1
IL10
LAG3
PDCD1
PDCD1LG2
TIGIT
TNFRSF9
Log2 expression
Tumor Inflammation Signature score

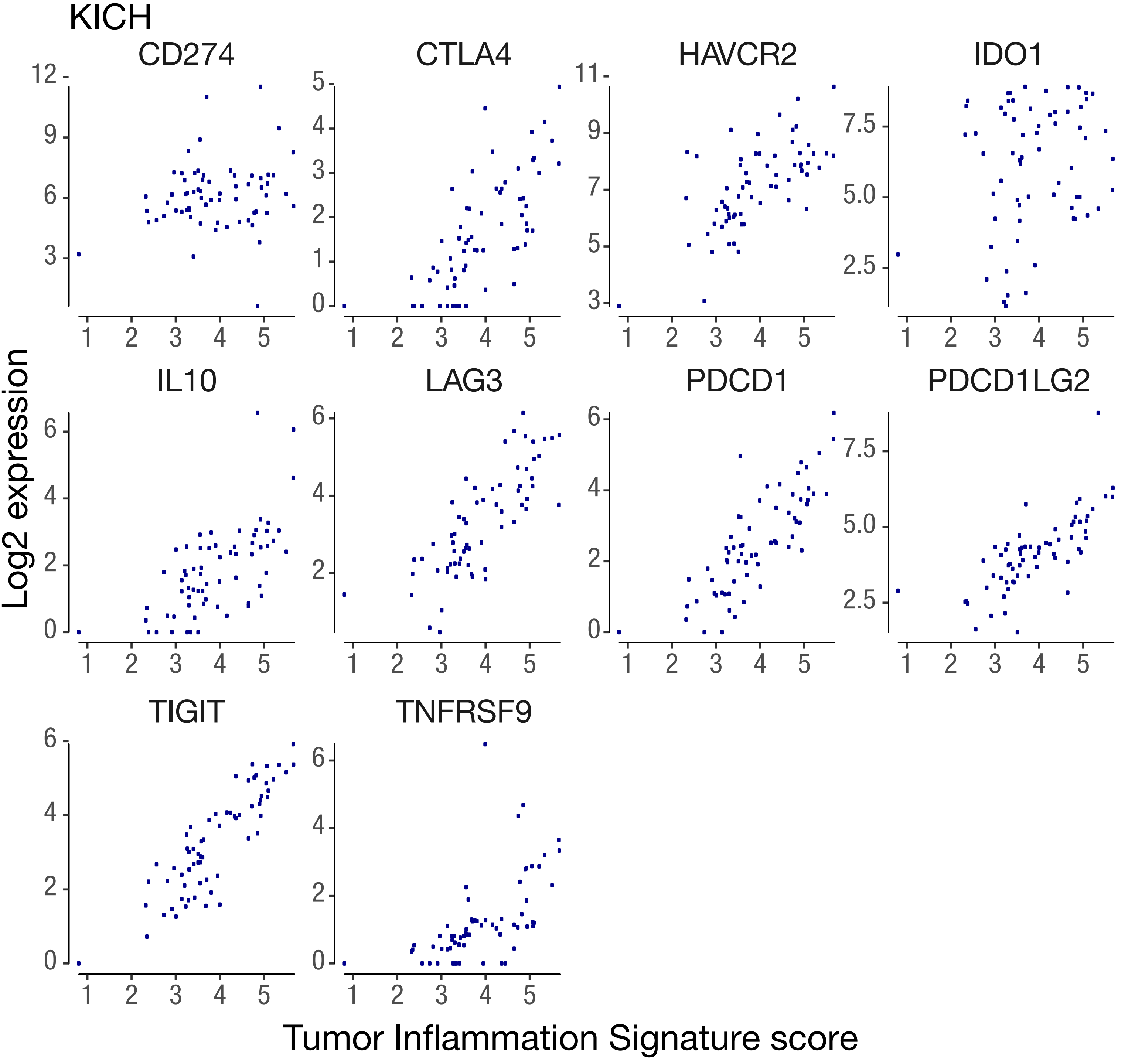
KICH
CD274
CTLA4
HAVCR2
IDO1
IL10
LAG3
PDCD1
PDCD1LG2
TIGIT
TNFRSF9
Log2 expression
Tumor Inflammation Signature score

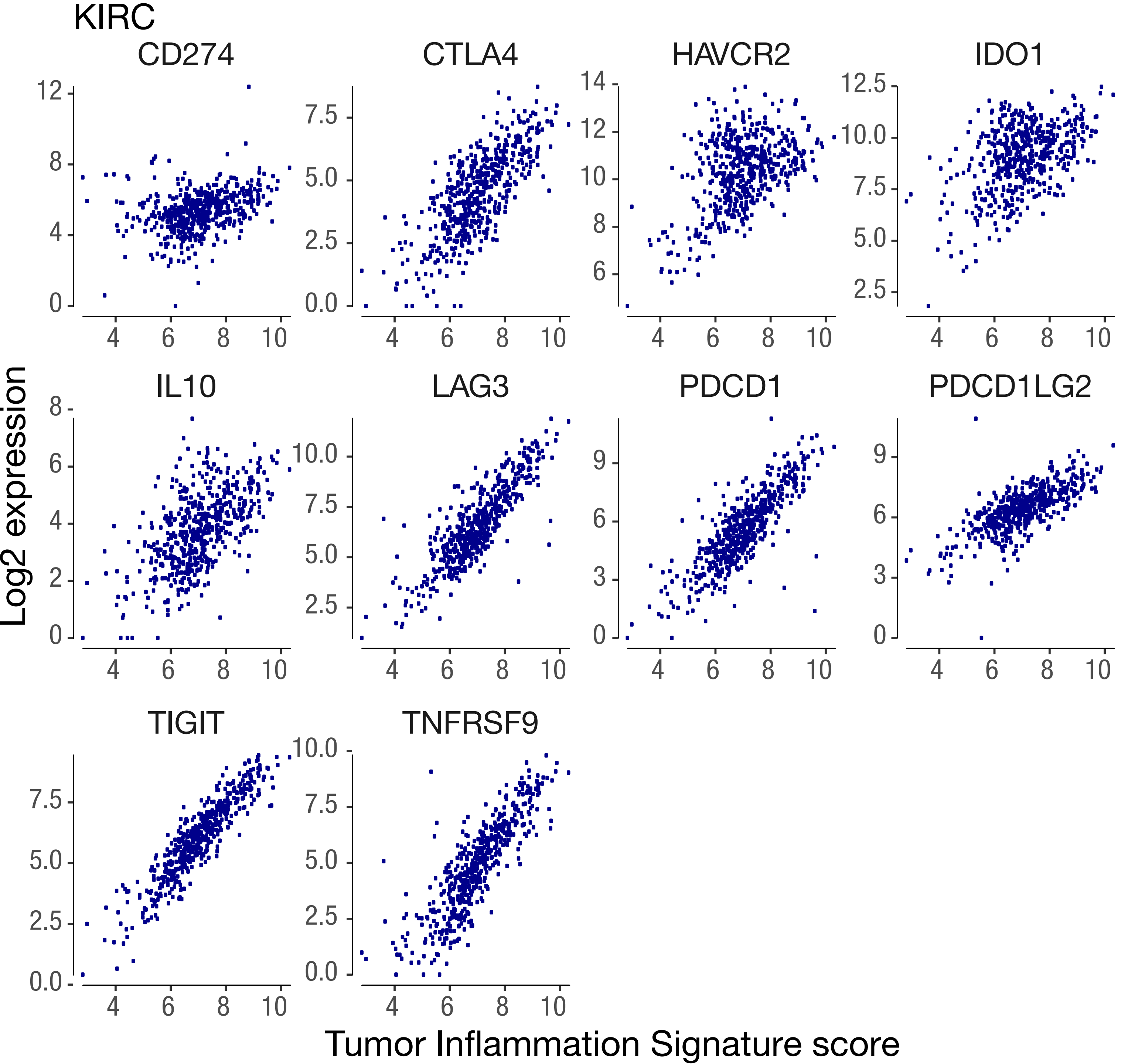
KIRC
CD274
CTLA4
HAVCR2
IDO1
IL10
LAG3
PDCD1
PDCD1LG2
TIGIT
TNFRSF9
Log2 expression
Tumor Inflammation Signature score

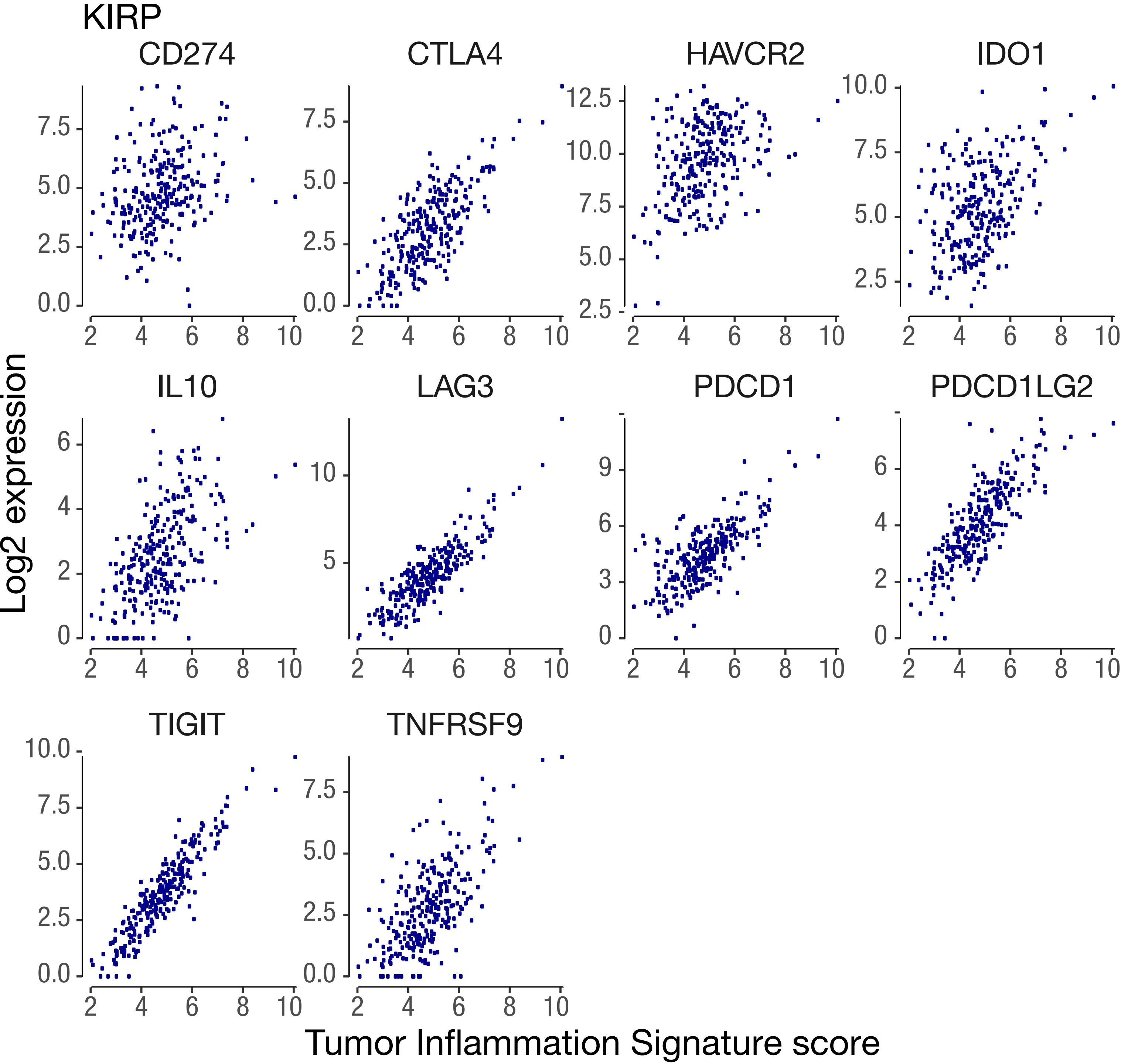
KIRP
CD274
CTLA4
HAVCR2
IDO1
IL10
LAG3
PDCD1
PDCD1LG2
TIGIT
TNFRSF9
Log2 expression
Tumor Inflammation Signature score

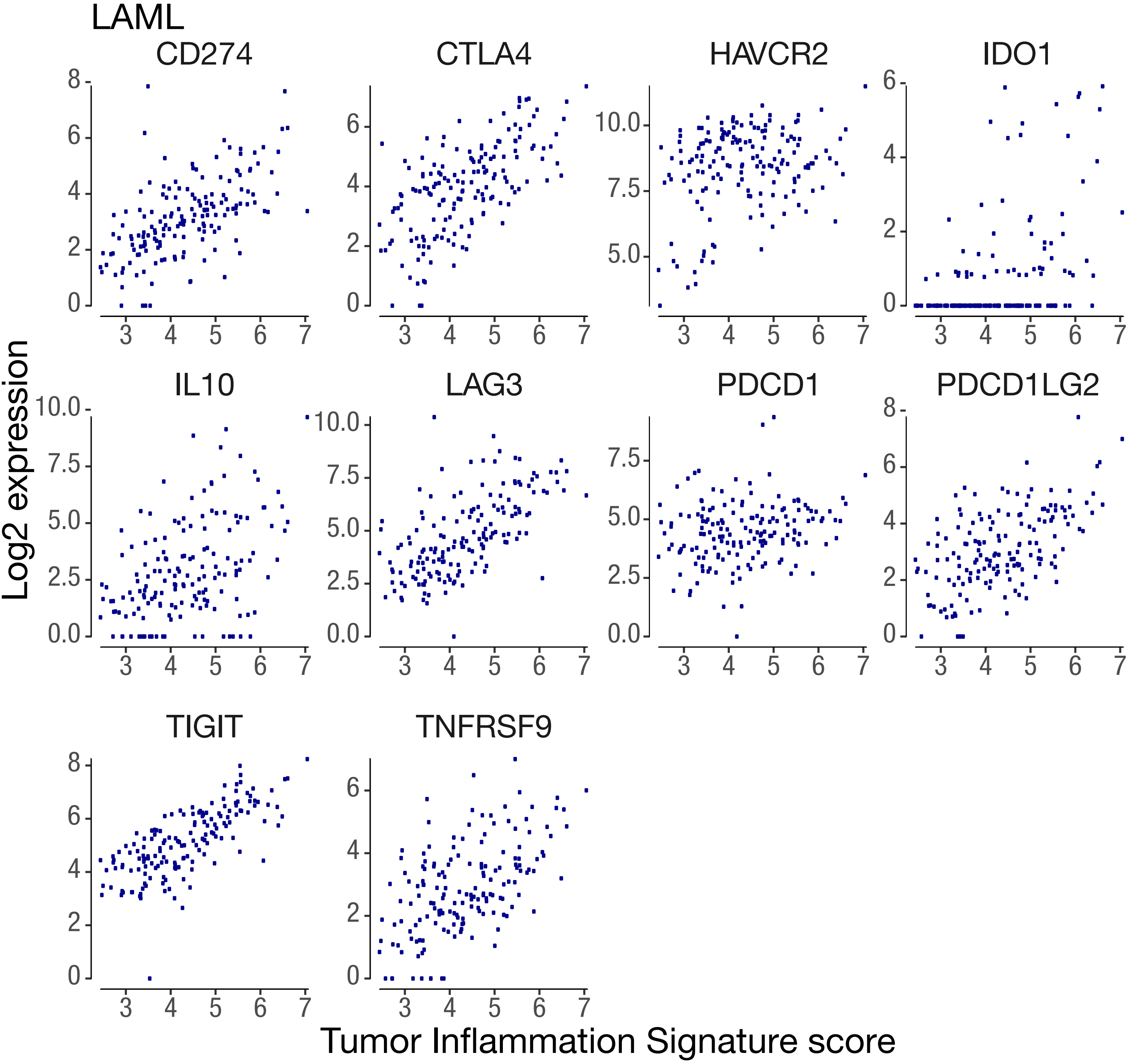

LAML
CD274
CTLA4
HAVCR2
IDO1
IL10
LAG3
PDCD1
PDCD1LG2
TIGIT
TNFRSF9
Log2 expression
Tumor Inflammation Signature score

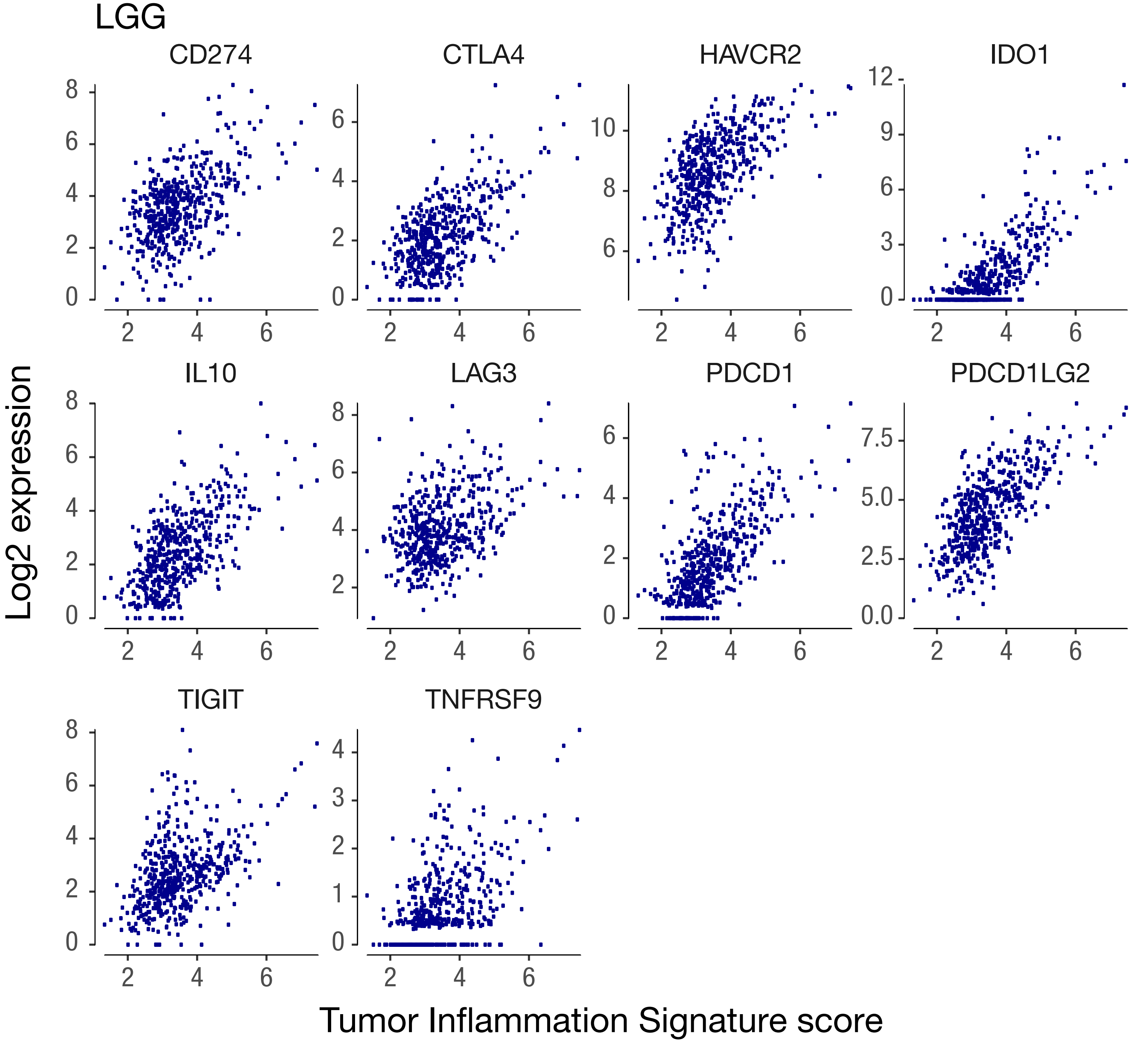
LGG
CD274
CTLA4
HAVCR2
IDO1
IL10
LAG3
PDCD1
PDCD1LG2
TIGIT
TNFRSF9
Log2 expression
Tumor Inflammation Signature score

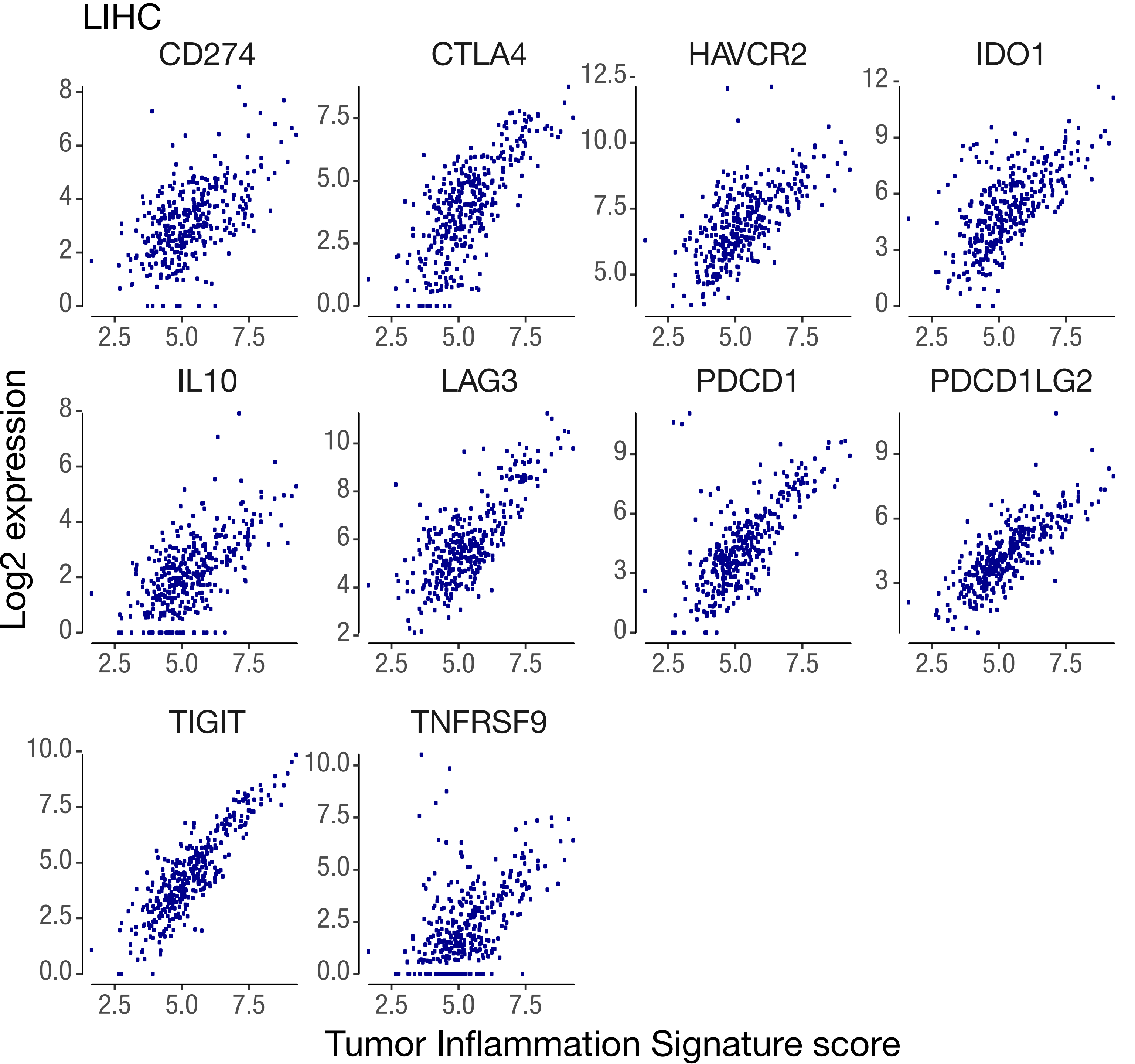

LIHC
CD274
CTLA4
HAVCR2
IDO1
IL10
LAG3
PDCD1
PDCD1LG2
TIGIT
TNFRSF9
Log2 expression
Tumor Inflammation Signature score

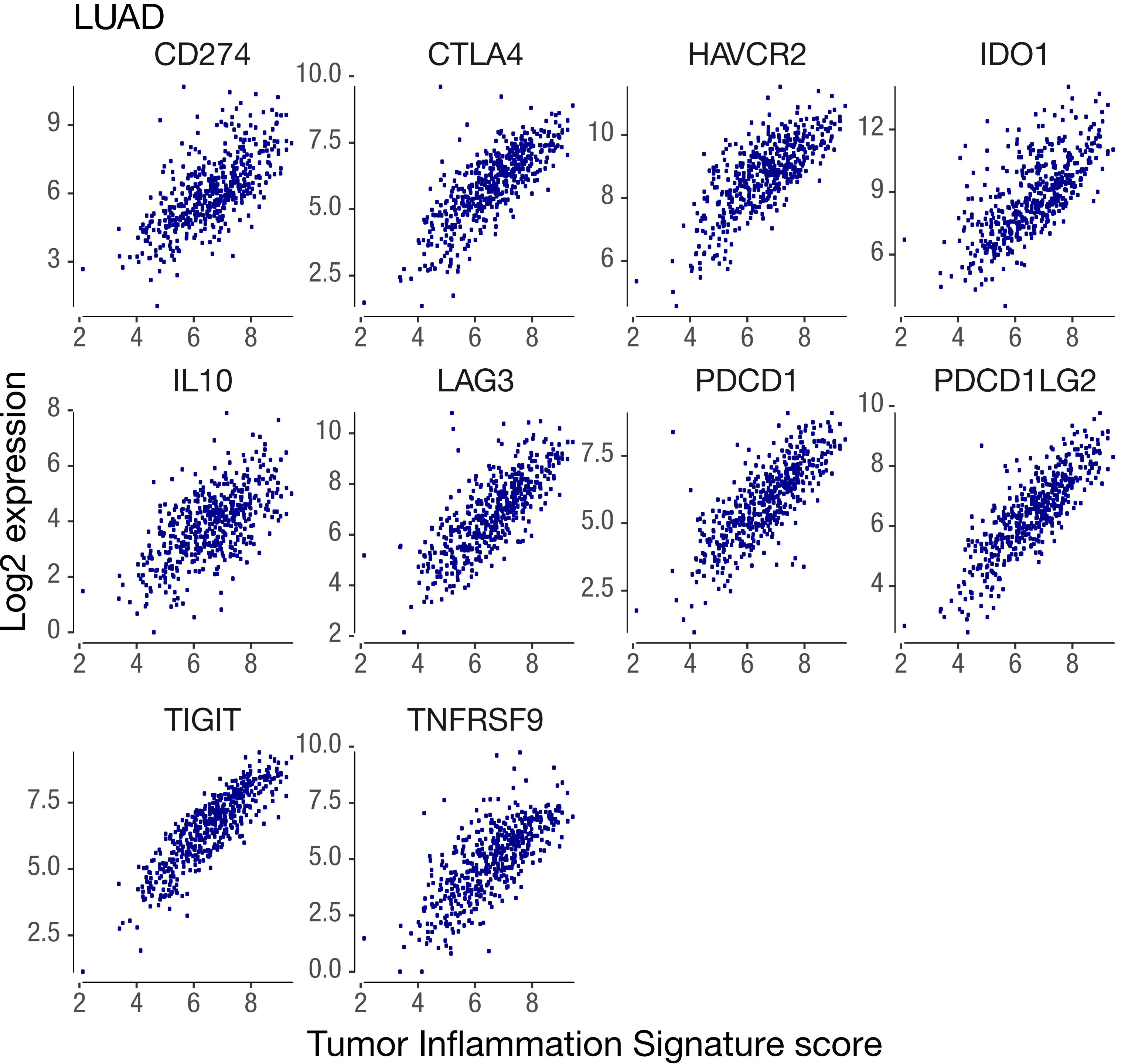

LUAD
CD274
CTLA4
HAVCR2
IDO1
IL10
LAG3
PDCD1
PDCD1LG2
TIGIT
TNFRSF9
Log2 expression
Tumor Inflammation Signature score

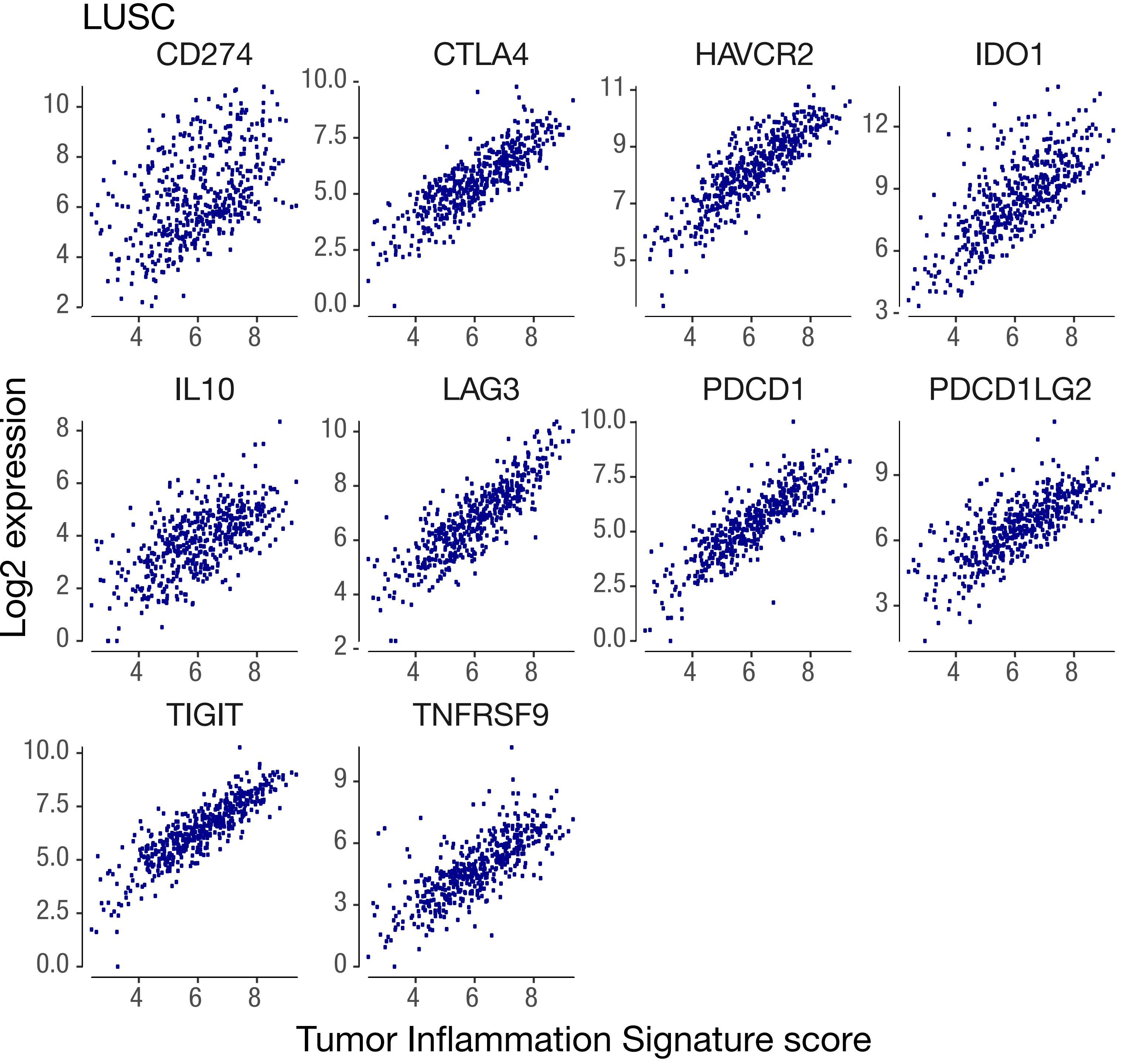
LUSC
CD274
CTLA4
HAVCR2
IDO1
IL10
LAG3
PDCD1
PDCD1LG2
TIGIT
TNFRSF9
Log2 expression
Tumor Inflammation Signature score

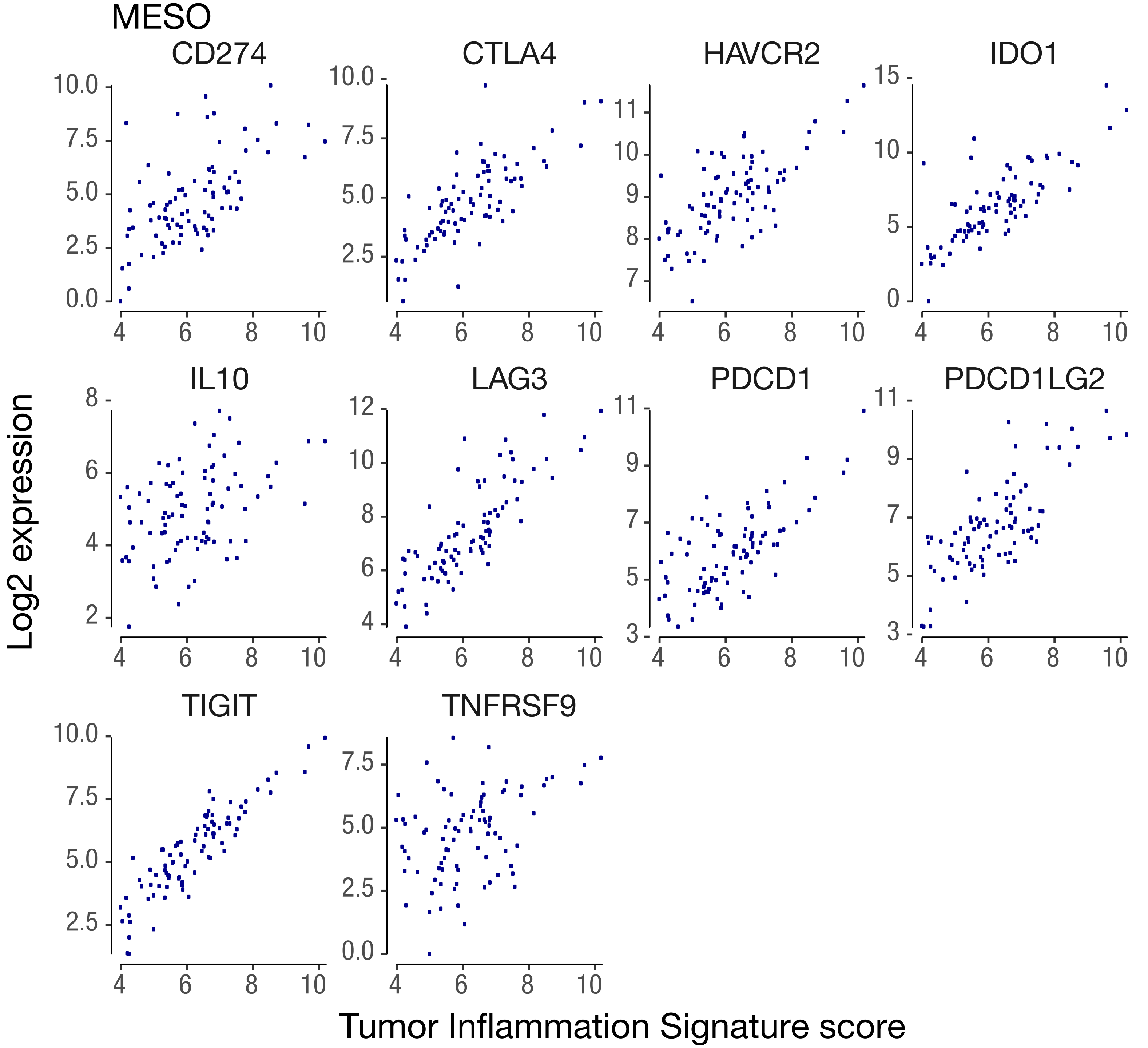
MESO
CD274
CTLA4
HAVCR2
IDO1
IL10
LAG3
PDCD1
PDCD1LG2
TIGIT
TNFRSF9
Log2 expression
Tumor Inflammation Signature score

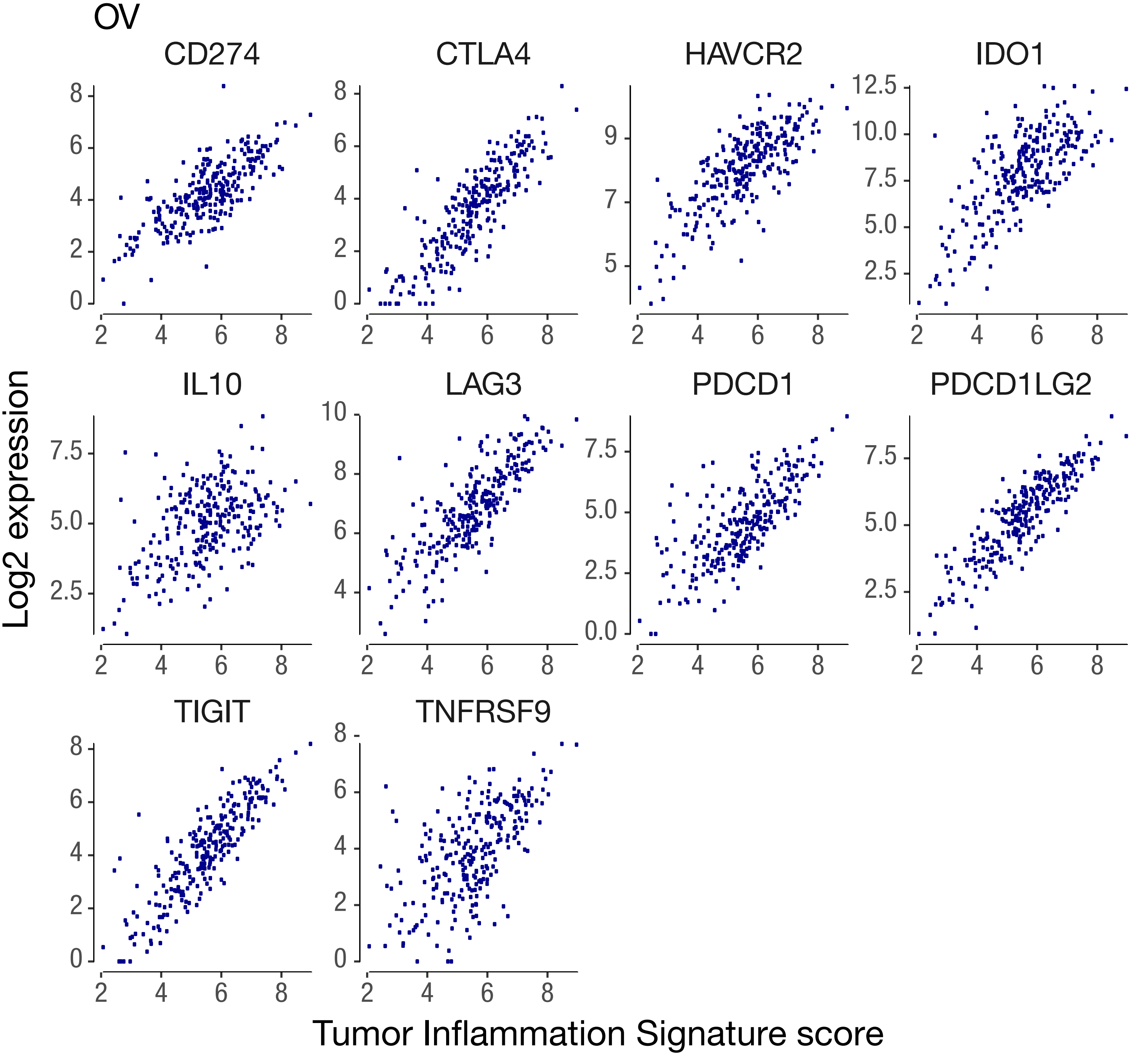
OV
CD274
CTLA4
HAVCR2
IDO1
IL10
LAG3
PDCD1
PDCD1LG2
TIGIT
TNFRSF9
Log2 expression
Tumor Inflammation Signature score

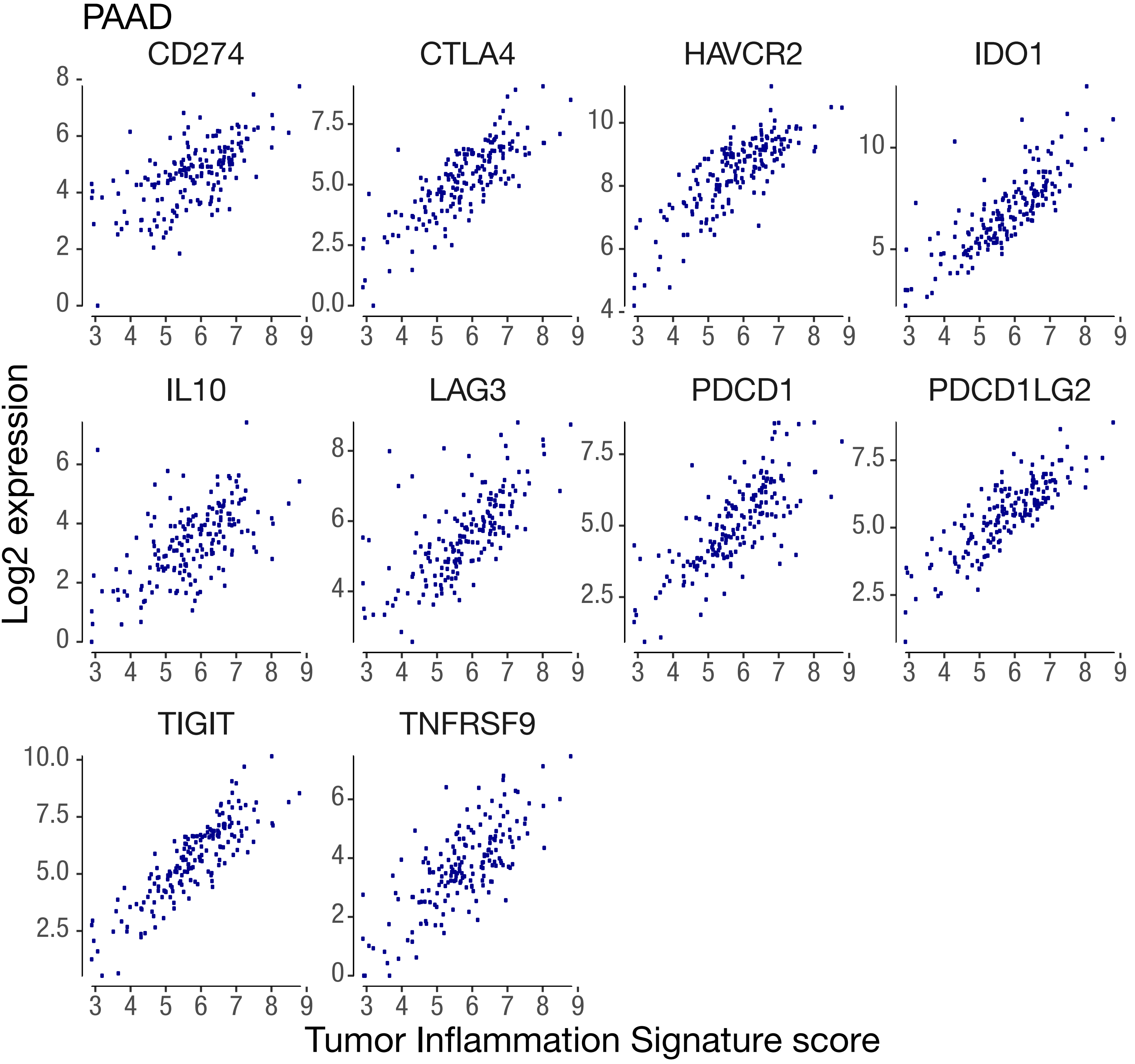
PAAD
CD274
CTLA4
HAVCR2
IDO1
IL10
LAG3
PDCD1
PDCD1LG2
TIGIT
TNFRSF9
Log2 expression
Tumor Inflammation Signature score

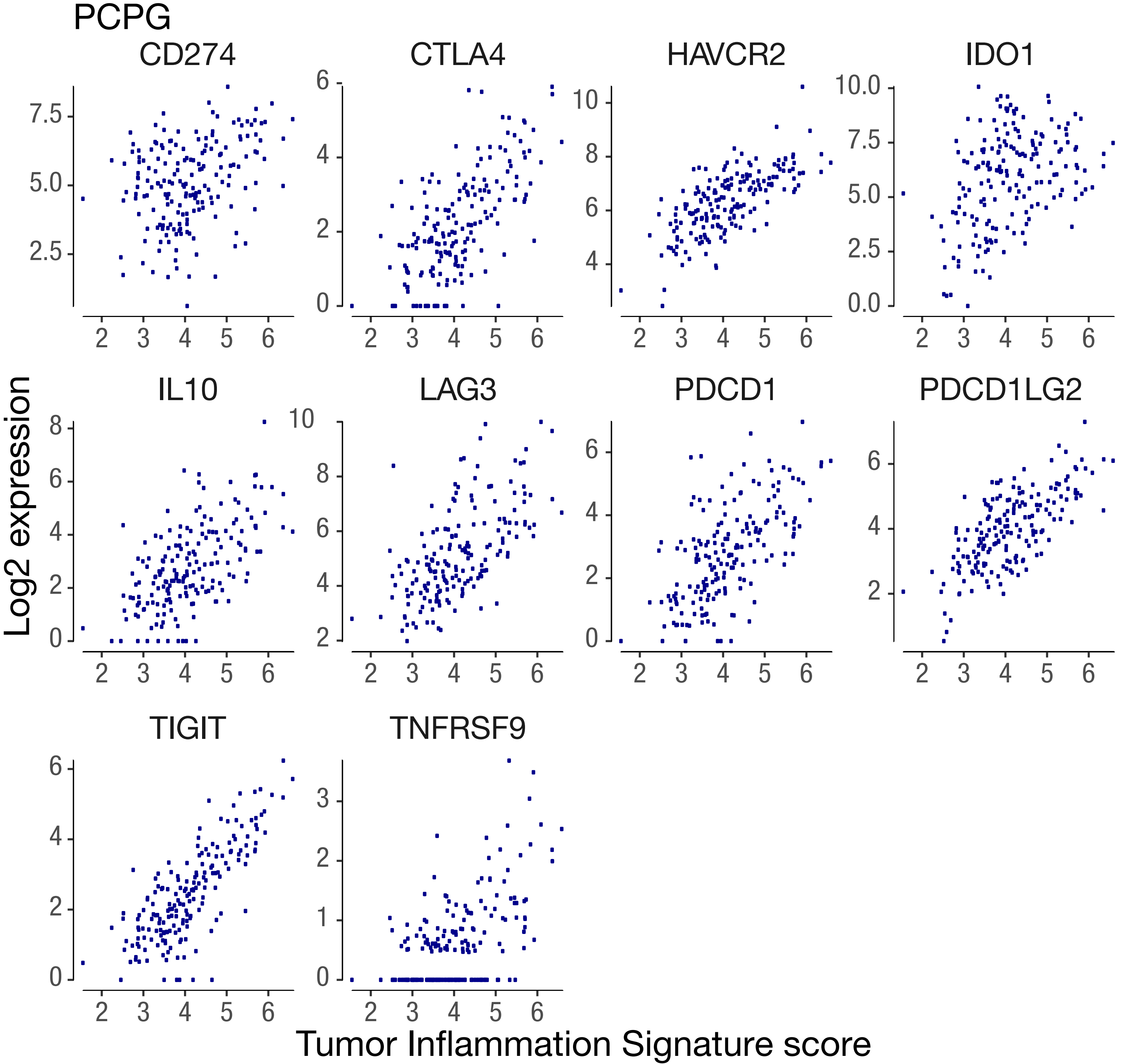
PCPG
CD274
CTLA4
HAVCR2
IDO1
IL10
LAG3
PDCD1
PDCD1LG2
TIGIT
TNFRSF9
Log2 expression
Tumor Inflammation Signature score

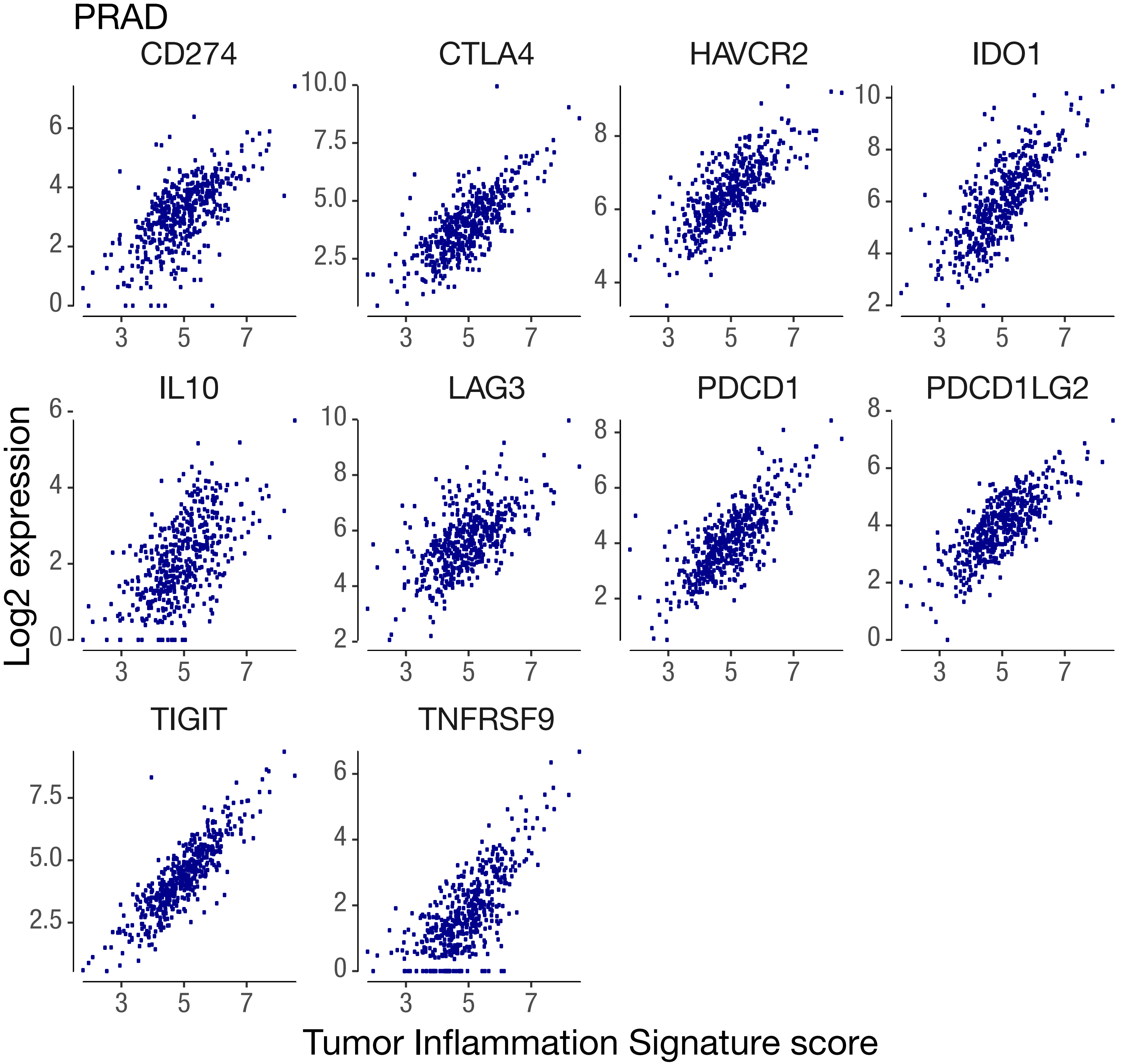
PRAD
CD274
CTLA4
HAVCR2
IDO1
IL10
LAG3
PDCD1
PDCD1LG2
TIGIT
TNFRSF9
Log2 expression
Tumor Inflammation Signature score

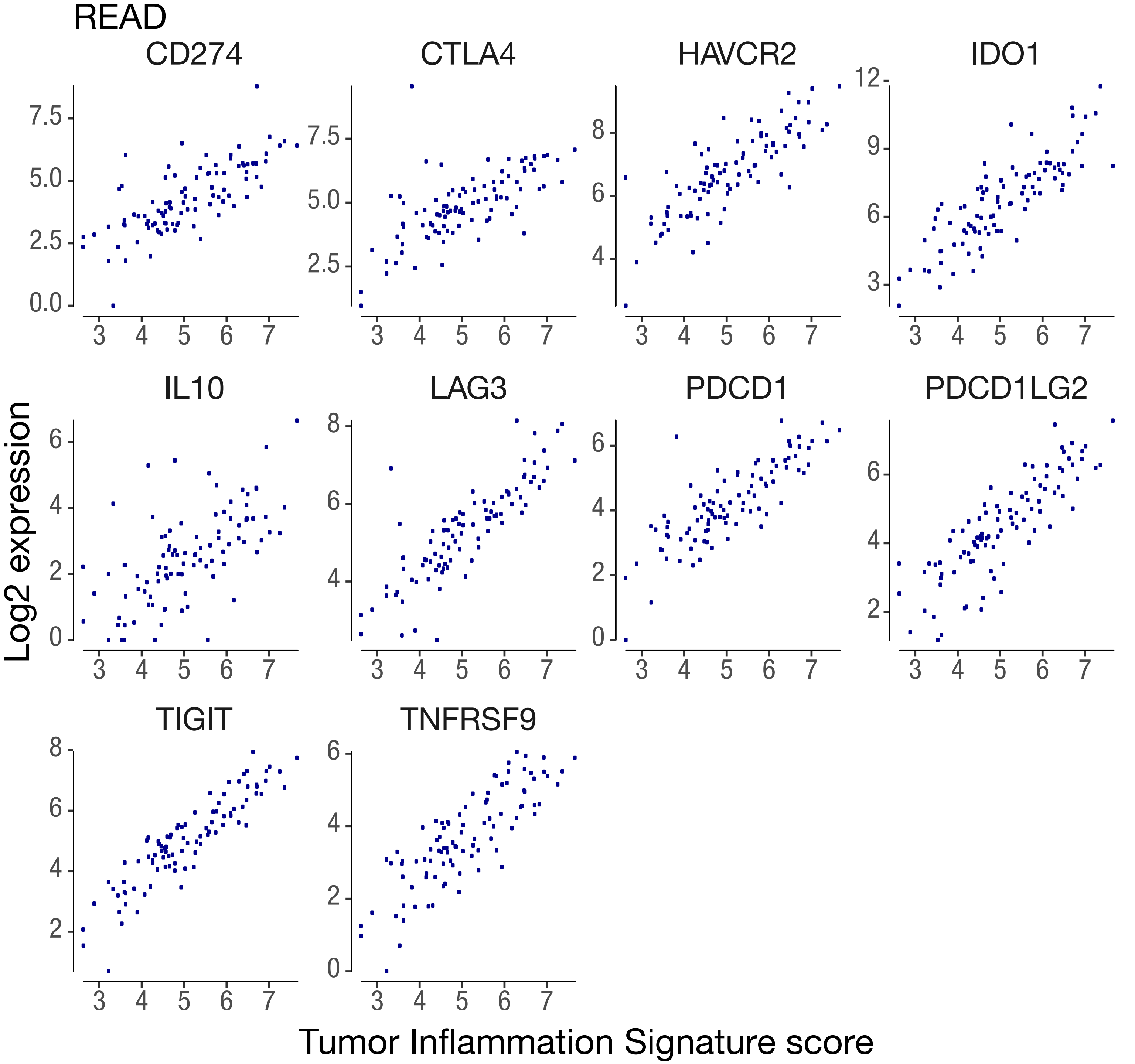
READ
CD274
CTLA4
HAVCR2
IDO1
IL10
LAG3
PDCD1
PDCD1LG2
TIGIT
TNFRSF9
Log2 expression
Tumor Inflammation Signature score

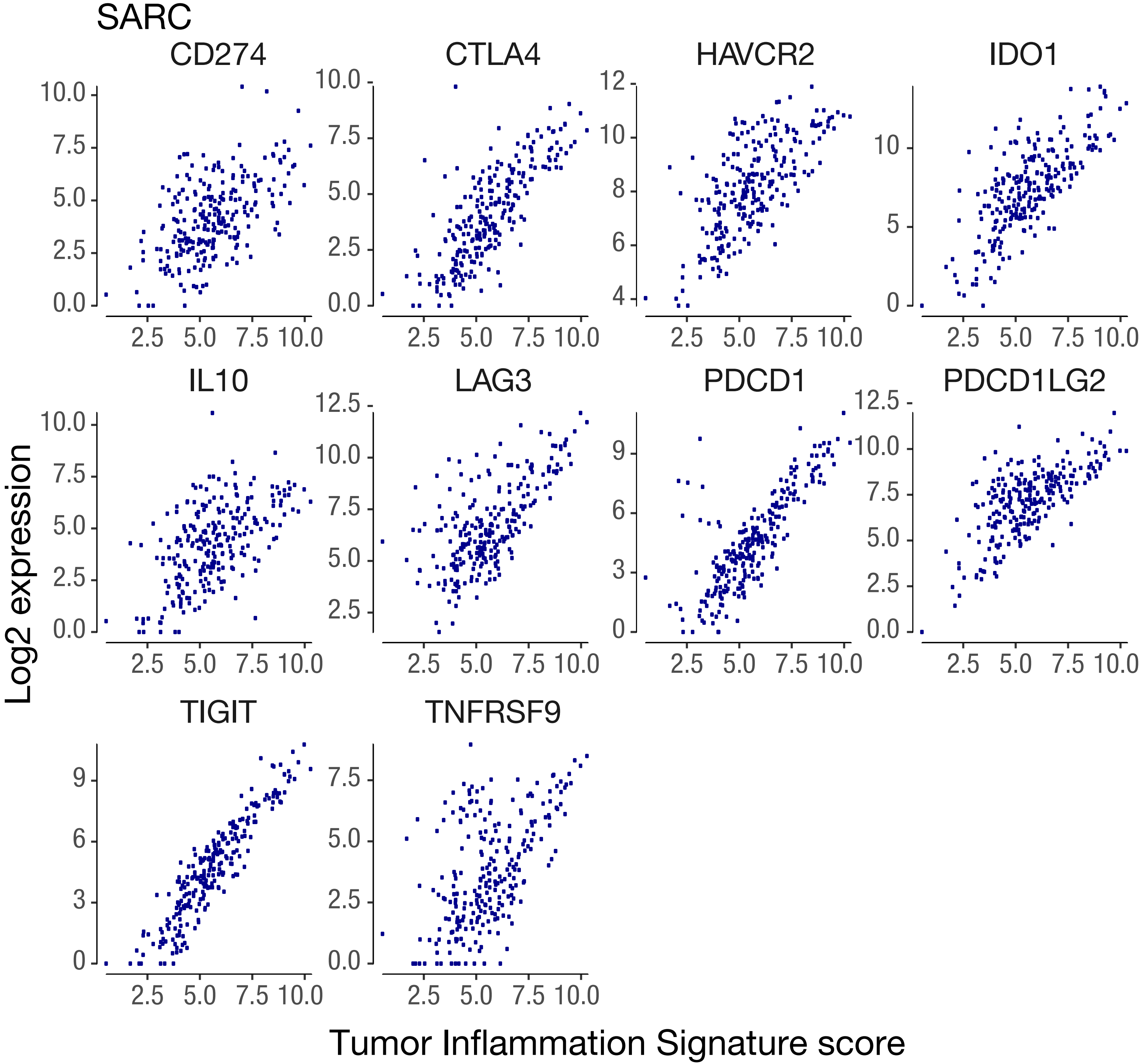

SARC
CD274
CTLA4
HAVCR2
IDO1
IL10
LAG3
PDCD1
PDCD1LG2
TIGIT
TNFRSF9
Log2 expression
Tumor Inflammation Signature score

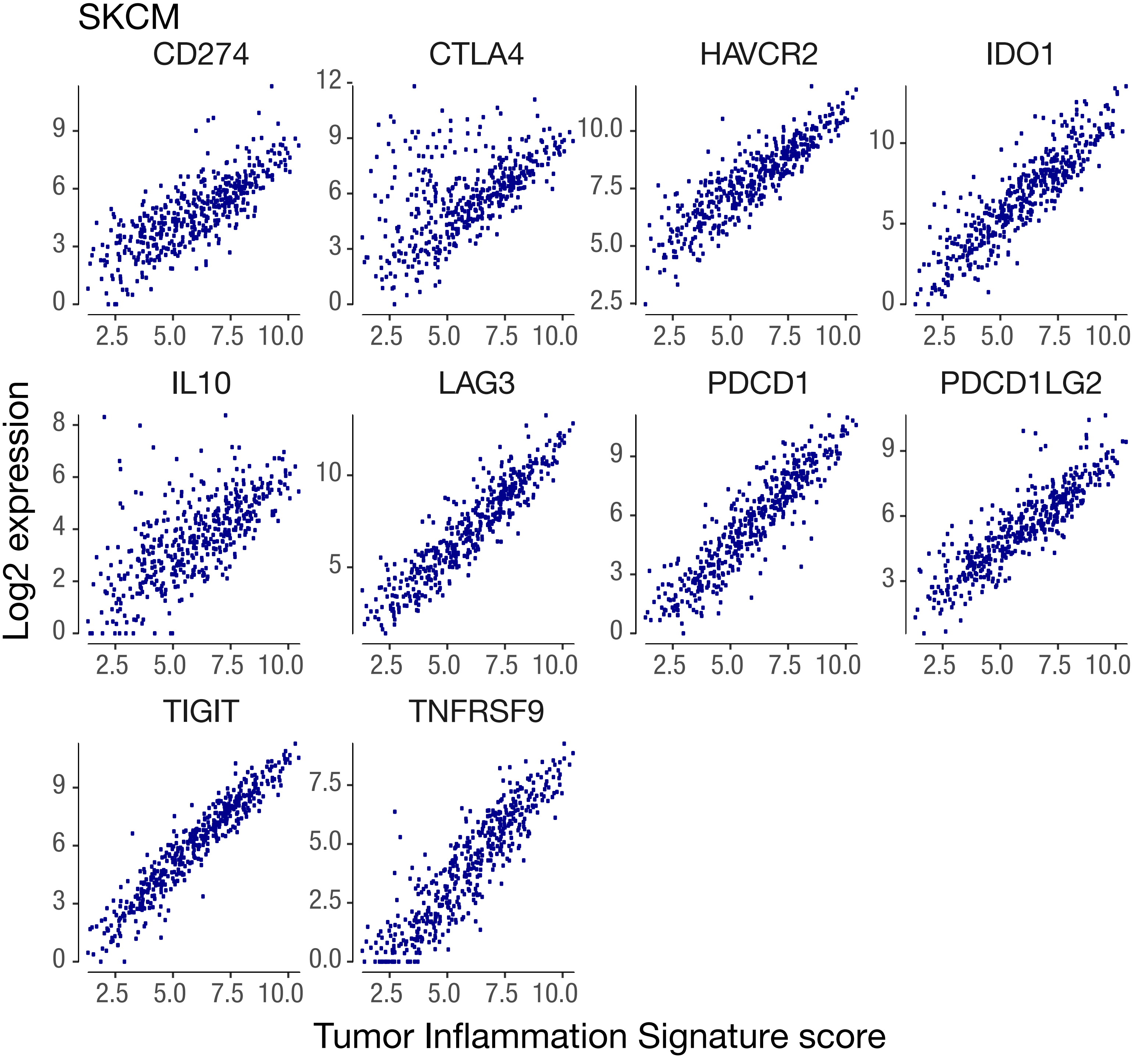
SKCM
CD274
CTLA4
HAVCR2
IDO1
IL10
LAG3
PDCD1
PDCD1LG2
TIGIT
TNFRSF9
Log2 expression
Tumor Inflammation Signature score

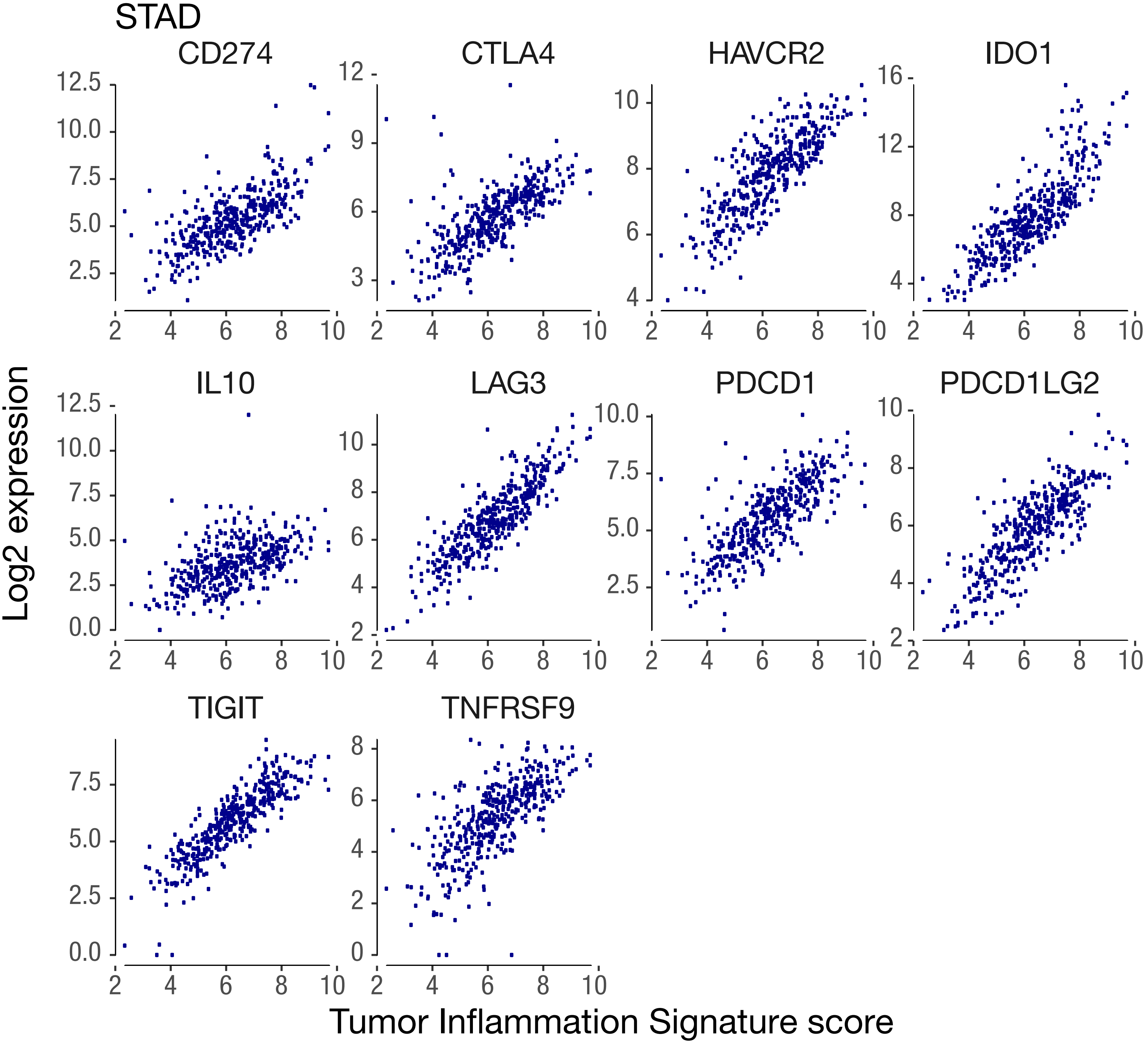
STAD
CD274
CTLA4
HAVCR2
IDO1
IL10
LAG3
PDCD1
PDCD1LG2
TIGIT
TNFRSF9
Log2 expression
Tumor Inflammation Signature score

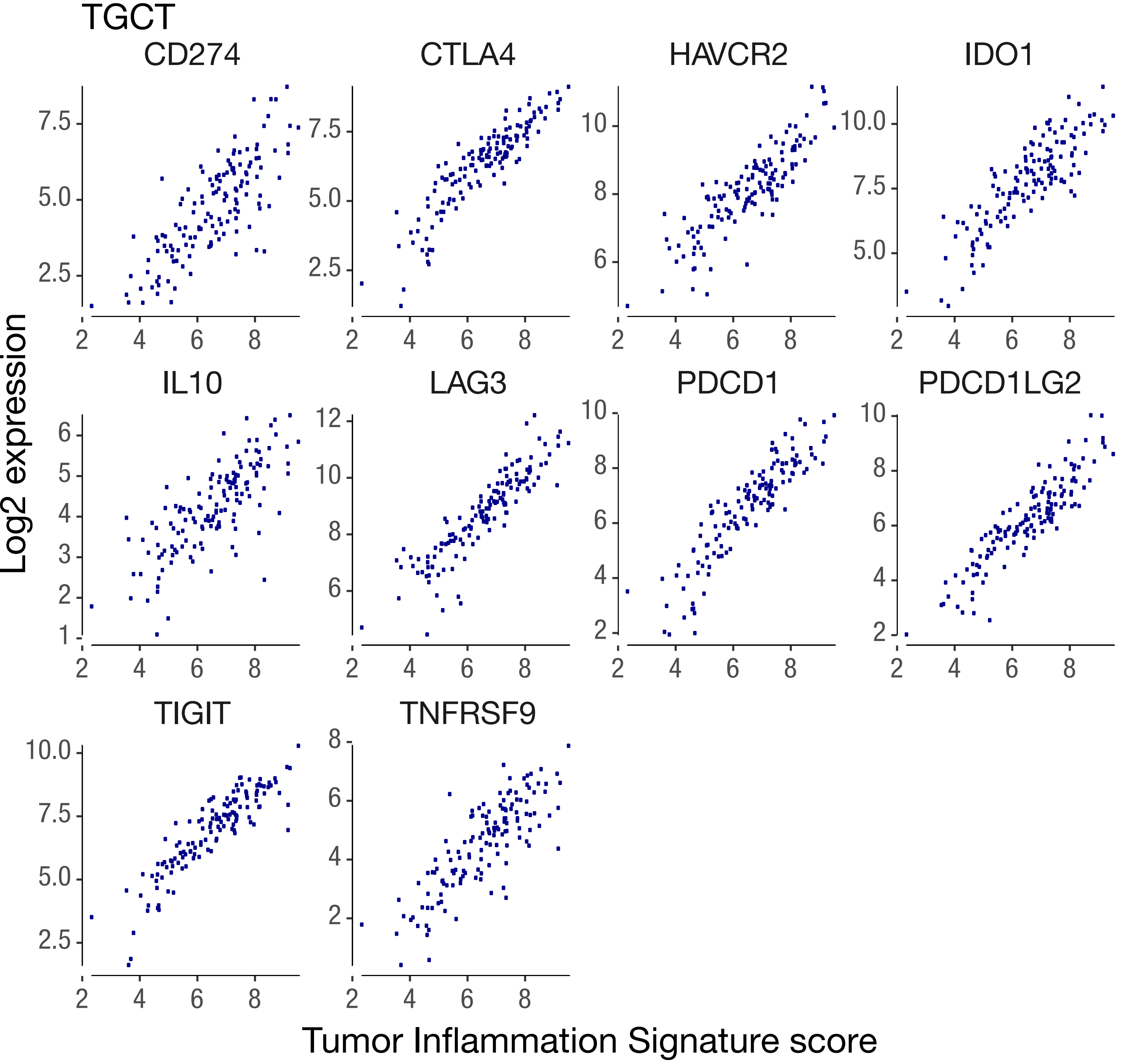

TGCT
CD274
CTLA4
HAVCR2
IDO1
IL10
LAG3
PDCD1
PDCD1LG2
TIGIT
TNFRSF9
Log2 expression
Tumor Inflammation Signature score

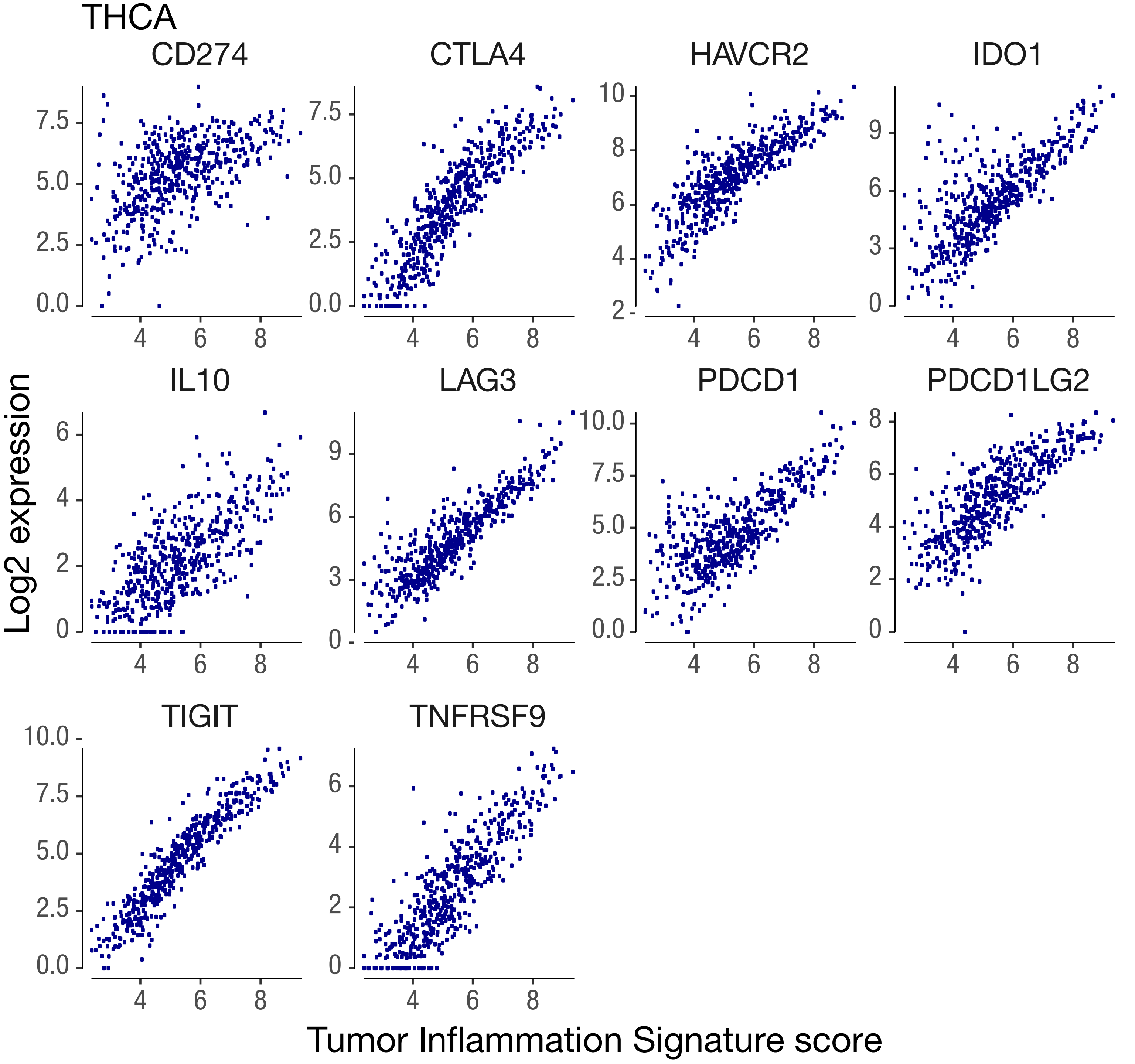
THCA
CD274
CTLA4
HAVCR2
IDO1
IL10
LAG3
PDCD1
PDCD1LG2
TIGIT
TNFRSF9
Log2 expression
Tumor Inflammation Signature score

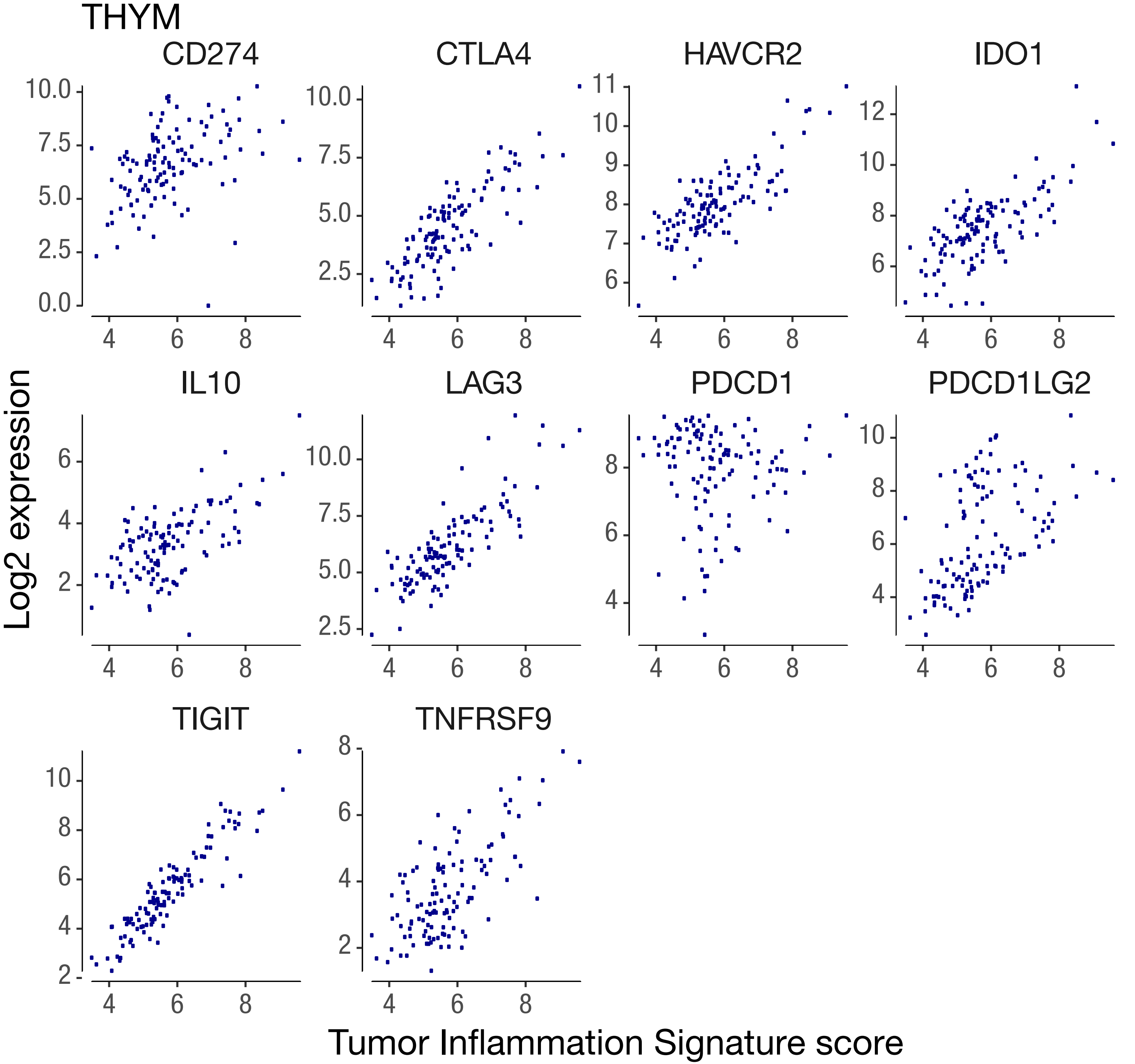
THYM
CD274
CTLA4
HAVCR2
IDO1
IL10
LAG3
PDCD1
PDCD1LG2
TIGIT
TNFRSF9
Log2 expression
Tumor Inflammation Signature score

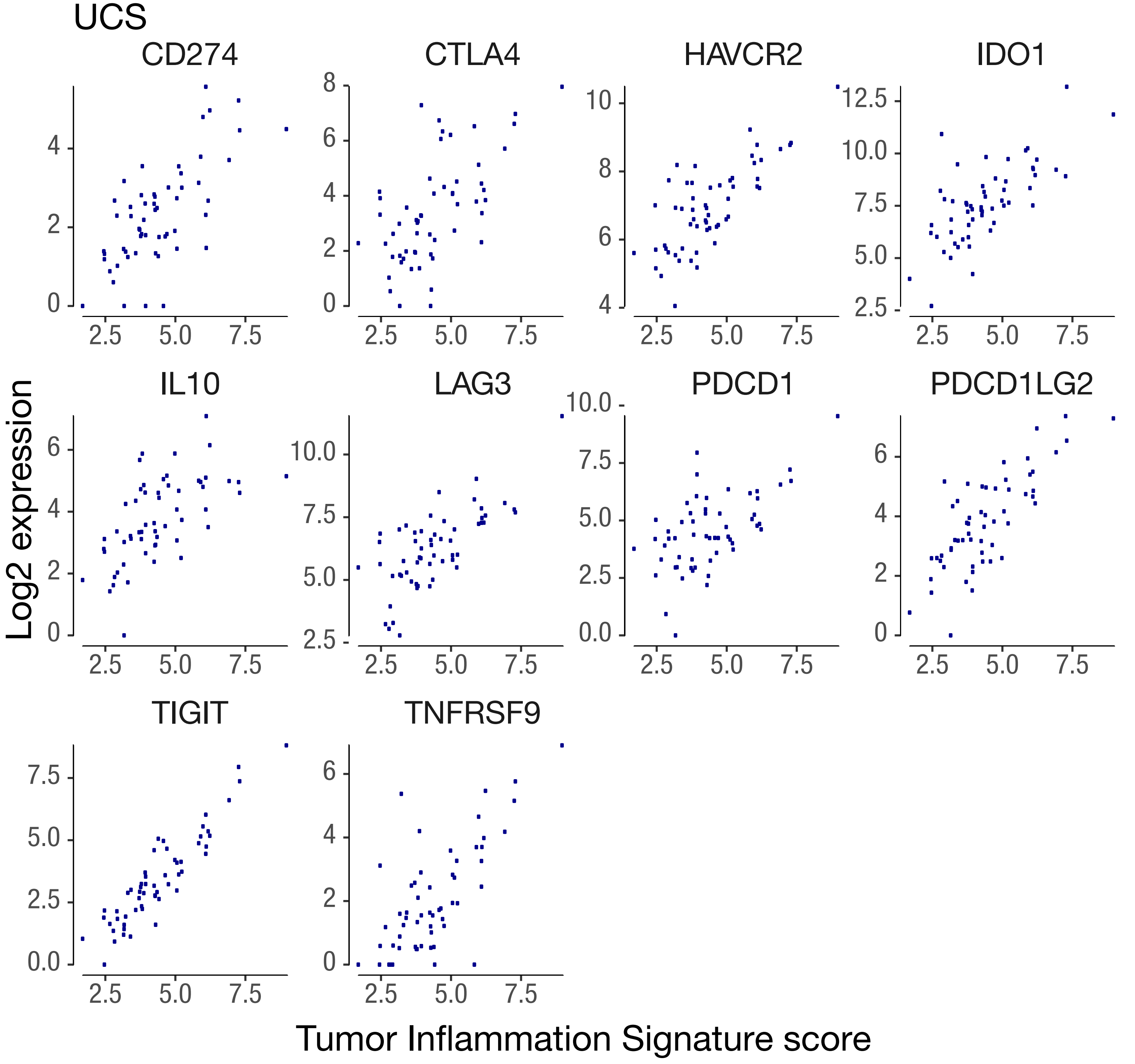
UCS
CD274
CTLA4
HAVCR2
IDO1
IL10
LAG3
PDCD1
PDCD1LG2
TIGIT
TNFRSF9
Log2 expression
Tumor Inflammation Signature score

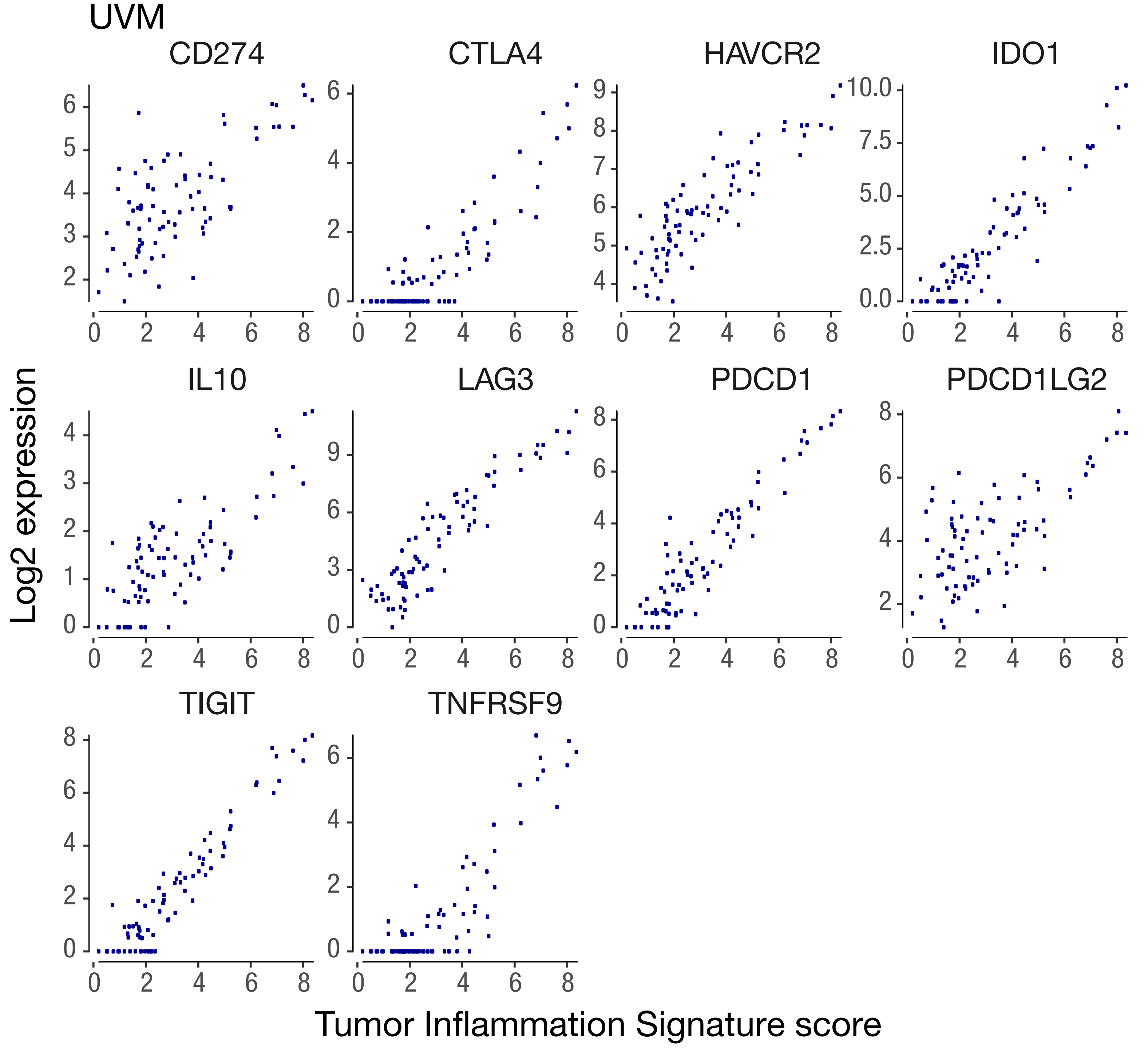

UVM
CD274
CTLA4
HAVCR2
IDO1
IL10
LAG3
PDCD1
PDCD1LG2
TIGIT
TNFRSF9
Log2 expression
Tumor Inflammation Signature score
